# Serotonergic neurons control cortical neuronal intracellular energy dynamics by modulating astrocyte-neuron lactate shuttle

**DOI:** 10.1016/j.isci.2022.105830

**Published:** 2023-01-05

**Authors:** Akiyo Natsubori, Shinobu Hirai, Soojin Kwon, Daisuke Ono, Fei Deng, Jinxia Wan, Momoka Miyazawa, Takashi Kojima, Haruo Okado, Akihiro Karashima, Yulong Li, Kenji F. Tanaka, Makoto Honda

**Affiliations:** 1 Sleep Disorders Project, Tokyo Metropolitan Institute of Medical Science, Setagaya-ku, Tokyo 156-8506, Japan; 2 Department of Neuroscience Ⅱ, Research Institute of Environmental Medicine, Nagoya University, Nagoya 464-8601, Japan; 3 Department of Neural Regulation, Nagoya University Graduate School of Medicine, Nagoya 466-8550, Japan; 4 State Key Laboratory of Membrane Biology, Peking University School of Life Sciences, Beijing 100871, China; 5 PKU-IDG/McGovern Institute for Brain Research, Beijing 100871, China; 6 Faculty of Science Division Ⅱ, Tokyo University of Science, Shinjuku-ku, Tokyo 162-8601, Japan; 7 Department of Electronics, Graduate School of Engineering, Tohoku Institute of Technology, Sendai 982-8577, Japan; 8 Division of Brain Sciences, Institute for Advanced Medical Research, Keio University School of Medicine, Shinjuku-ku, Tokyo 160-8582, Japan

**Keywords:** Biological sciences, Neuroscience, molecular neuroscience

## Abstract

The central serotonergic system has multiple roles in animal physiology and behavior, including sleep-wake control. However, its function in controlling brain energy metabolism according to the state of animals remains undetermined. Through *in vivo* monitoring of energy metabolites and signaling, we demonstrated that optogenetic activation of raphe serotonergic neurons increased cortical neuronal intracellular concentration of ATP, an indispensable cellular energy molecule, which was suppressed by inhibiting neuronal uptake of lactate derived from astrocytes. Raphe serotonergic neuronal activation induced cortical astrocytic Ca^2+^ and cAMP surges and increased extracellular lactate concentrations, suggesting the facilitation of lactate release from astrocytes. Furthermore, chemogenetic inhibition of raphe serotonergic neurons partly attenuated the increase in cortical neuronal intracellular ATP levels as arousal increased in mice. Serotonergic neuronal activation promoted an increase in cortical neuronal intracellular ATP levels, partly mediated by the facilitation of the astrocyte-neuron lactate shuttle, contributing to state-dependent optimization of neuronal intracellular energy levels.

## Highlights

• Raphe serotonergic neurons (STn) enhance cortical neuronal intracellular ATP levels
• STn induces cortical astrocytic cAMP and Ca^2+^ surges and promotes ANLS
• STn-induced cortical neuronal ATP increase attenuates by blocking ANLS
• STn inhibition attenuates cortical neuronal ATP increase in awakening from REM sleep

## Graphical abstract

**Figure:**
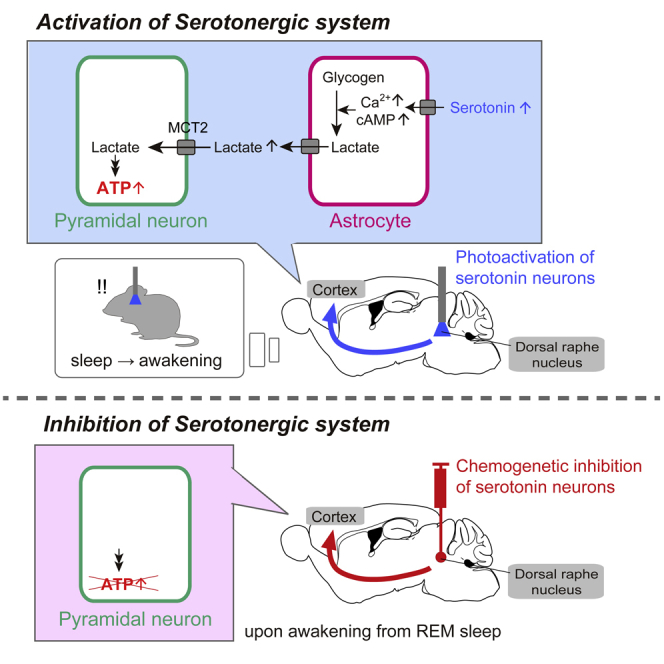

## Introduction

The central serotonergic system is crucially involved in a wide range of brain functions such as mood, memory, feeding, and sleep-wake control.^1^^,^^2^^,^^3^^,^^4^ The serotonergic neurons, which mainly originate in the raphe nuclei, project to almost all brain regions, including throughout the cortex, and in those places, diffuse serotonin by volume transmission.^5^^,^^6^ The serotonergic neurons located in the dorsal raphe nucleus change their firing pattern depending on the sleep-wake state of animals; burst firing causes the waking of animals from sleep.^4^ Although the central serotonergic system can increase arousal in animals, it has not been clarified whether it has a function to control cerebral energy metabolism to adapt to the increased neuronal energy demand with the animals’ arousal increase.

To adjust neuronal energy levels to their demand, the local brain hemodynamics associated with neuronal activity, known as neurovascular coupling, could be regarded as a mechanism of local brain energy homeostasis.^7^^,^^8^ Furthermore, a coordinated brain-wide energy metabolism regulation mechanism corresponding to the states of animals has been assumed, because brain metabolic activities and neuronal firing activities synchronously fluctuate in a broad area of the brain depending on the vigilance state of animals.^9^^,^^10^ Before the exploration of brain-wide energy homeostatic mechanisms, we previously clarified the outcome of these brain energy homeostatic mechanisms in terms of the dynamics of neuronal intracellular concentrations of adenosine 5-triphosphate (ATP), a major cellular energy metabolite.^11^^,^^12^ Previous *in vitro* study has demonstrated that neuronal intracellular ATP concentrations directly reflect neuronal energy synthesis and consumption,^13^ which can be regarded as the cellular energy status.^12^ Using a genetically encoded fluorescent ATP sensor,^14^ we demonstrated that the intracellular concentrations of ATP in cortical pyramidal neurons fluctuated synchronously throughout the cortex depending on the vigilance state of mice, and importantly, always promptly increased as arousal levels increased in mice.^12^ This suggests that integrated mechanisms of cortex-wide regulation of energy metabolism could facilitate neuronal ATP synthesis, exceeding its increased energy demand throughout the cortex in accordance with the arousal increase in mice.

In this study, we hypothesized that the central serotonergic system is involved in the regulation of brain energy metabolism, resulting in state-dependent cortex-wide neuronal intracellular ATP dynamics. This is supported by the anatomical and functional features of raphe serotonergic neurons, namely the widespread projection from the raphe nuclei to almost all brain regions and state-dependent firing activities.^4^^,^^5^ In addition to cortical neurons, astrocytes, a type of glial cell that functions to adjust local brain energy metabolism, have been reported to be regulated in their various activities by serotonin via several receptor subtypes.^15^^,^^16^^,^^17^ This suggests that the energy metabolic activities in both cell types could be under the control of the serotonergic system.

One of the main functions of astrocytes in energy metabolism is to synthesize lactate mainly from intracellular glycogen storage and transfer it to neighboring neurons, known as the astrocyte-neuron lactate shuttle (ANLS).^18^ As multiple neurotransmitters, such as noradrenaline and glutamate, have been reported to facilitate lactate synthesis in astrocytes and its transfer to neurons,^18^ serotonin might also play a role in the regulation of ANLS. In turn, the lactate taken up into the neurons could provide a priority shortcut to the major energy metabolic route for ATP synthesis consisting of glycolysis, TCA cycle, and oxidative phosphorylation in neurons,^18^^,^^19^ which might also be under serotonergic control. In this study, we tested the hypothesis that raphe serotonergic neuronal activity increases cortical neuronal intracellular ATP levels by facilitating ANLS, which contributes to state-dependent neuronal intracellular energy dynamics.^12^

## Results

### Optogenetic activation of raphe serotonergic neurons increases cortical neuronal intracellular ATP levels

**Figure 1:**
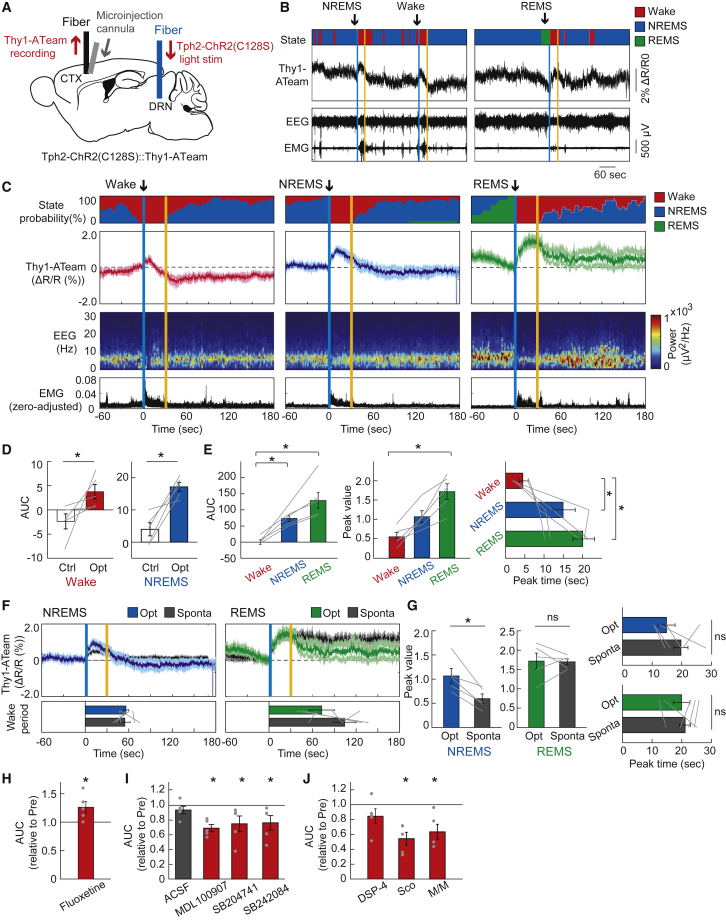
Response of cortical neuronal intracellular ATP signals to optogenetic activation of raphe serotonergic neurons (A) Schematic illustration of the fiber photometric recording of intracellular ATP levels in cortical pyramidal neurons under photostimulation of serotonergic neurons in the raphe. CTX, cortex; DRN, dorsal raphe nucleus. (B) Representative traces of cortical neuronal intracellular ATP signals (Thy1-ATeam), EEG, and EMG signals with vigilance states of animals under serotonergic photostimulation. One-second blue light illumination for the opening of ChR2(C128S) in serotonergic neurons was followed by 1-s yellow light illumination for the closing of ChR2(C128S), with a 30-s interval between illuminations. Vertical blue and yellow lines indicate the 1-s illumination of each light color. (C) Traces of averaged Thy1-ATeam signals with state probabilities, EEG power density spectrum, and EMG activity under serotonergic photostimulation during wake, NREM sleep, and REM sleep states, respectively. Traces of Thy1-ATeam signals represent mean ± SEM (n = 5 sessions from 5 mice). (D) Area under the curve (AUC) of Thy1-ATeam signal responses to the optogenetic activation of serotonergic neurons. ∗p< 0.05 versus Control (Ctrl); two-sided Wilcoxon signed-rank test (n = 5 sessions from 5 mice). In the control condition, 1-s yellow light illumination was used instead of blue light. (E) Comparison of the AUC, peak value, and the peak time of Thy1-ATeam signal responses to serotonergic activation across the states for the data in (C). ∗p< 0.05 versus Wake; Friedman test with *post hoc* Steel-Dwass test (n = 5 sessions from 5 mice). (F) Comparison of fluctuations in Thy1-ATeam signals under serotonergic photostimulation-induced awakening and spontaneous awakening from NREM sleep (left) and REM sleep state (right), respectively (n = 5 sessions from 5 mice). (G) Comparison of the peak values and peak times of Thy1-ATeam signals under serotonergic photostimulation-induced and spontaneous awakening for the data in (F). ∗p< 0.05 versus Sponta; two-sided Wilcoxon signed-rank test (n = 5 sessions from 5 mice). (H) The effect of fluoxetine on the AUC of Thy1-ATeam signal responses to serotonergic photostimulation. ∗p< 0.05 versus Pre (before fluoxetine treatment); two-sided Wilcoxon signed-rank test (n = 5 sessions from 5 mice). (I) Effect of serotonin receptor subtype-selective antagonists on the AUC of Thy1-ATeam signal responses to serotonergic photostimulation. ∗p< 0.05 versus Pre (before each drug administration); two-sided Wilcoxon signed-rank test (n = 5 sessions from 5 mice). (J) The effect of selective noradrenergic lesions by DSP-4 (left) and muscarinic and nicotinic cholinergic receptor antagonists (scopolamine (Sco) and mecamylamine hydrochloride/methyllycaconitine citrate (M/M), respectively; right) on the AUC of Thy1-ATeam signal responses to serotonergic photostimulation. *p*< 0.05 versus Pre (before each drug administration); two-sided Wilcoxon signed-rank test (n = 5 sessions from 5 mice). Data are expressed as the mean ± SEM. See also Figures S1–S4.

To investigate the hypothesis that serotonergic neuronal activity in the raphe nucleus induces an increase in cortical neuronal intracellular ATP concentrations, we conducted *in vivo* fiber photometric measurements of cortical neuronal intracellular ATP levels under the optogenetic activation of raphe serotonergic neurons with *Tph2*-tTA::tetO-ChR2(C128S)::Thy1-ATeam triple-transgenic mice (Tph2-ChR2(C128S)::Thy1ATeam; Figure 1A). In this mouse line, the axon terminals of ChR2(C128S)-positive serotonergic neurons were distributed in layer 5 of the cerebral cortex, where ATeam-positive pyramidal neurons are expressed (Figure S1A). However, the continuous excitation light irradiation for ATeam in the cortex was not expected to be intense enough to stimulate the serotonergic terminals via ChR2(C128S).^20^

We observed that the intracellular ATP levels in cortical pyramidal neurons increased by optogenetic activation of raphe serotonergic neurons for 30 s during the wake, sleep, and anesthetized states in mice (Figures 1B, 1C, and S2). These serotonergic activation-induced increases in neuronal ATP levels were always accompanied by EEG/EMG changes, representing the arousal level increase in mice, consistent with previous reports that the burst firing of raphe serotonergic neurons induced the awakening of animals.^4^^,^^21^ Yellow light illumination as a control to the raphe nucleus did not induce Thy1-ATeam signal fluctuations (Figures 1D and S1C). In addition, in mice, serotonergic photostimulation and evoked arousal from sleep did not induce significant fluctuation of autofluorescence in the brain, ratio of mVenus and CFP-derived fluorescence, or ATeam mutant (AT1.03RK) signal (Figures S1D–S1F). The extent of neuronal intracellular ATP response differed depending on the state of the animals at the time of serotonergic photostimulation (Figures 1C, 1E, and S2). Serotonergic activation during the wake state induced the smallest increase in neuronal ATP levels, whereas serotonergic activation during the REM sleep state resulted in the largest increase. Under isoflurane anesthesia, serotonergic activation induced a slower increase in neuronal ATP levels compared with that during the non-REM sleep state (Figure S2). To clarify the reason for this state-dependent difference in neuronal ATP response, we monitored the dynamics of cortical extracellular serotonin concentration under serotonergic photostimulation using a genetically encoded fluorescent sensor, GRAB_5-HT_.^22^ We injected an adeno-associated virus (AAV) expressing GRAB_5-HT2h_ with the human synapsin (hSyn) promoter into the cortex of Tph2-ChR2(C128S) mice and measured extracellular serotonin signals using fiber photometry (Figure S3A). We observed a small increase in cortical extracellular serotonin signals by raphe serotonergic photostimulation for 30 s during the wake state, whereas a larger increase was observed during the sleep state (Figures S3B and S3C), which was comparable to the trend observed in neuronal ATP signal responses. Yellow light illumination as a control to the raphe nucleus did not induce hSyn-GRAB_5-HT2h_ signal fluctuation, and serotonergic photostimulation and evoked arousal from non-REM sleep in mice did not increase hSyn-GFP-derived fluorescence intensity as another control (Figures S3D–S3G). Under isoflurane anesthesia, the cortical extracellular serotonin dynamics by raphe serotonergic photostimulation seem to have the same trend as the neuronal ATP response, which is delayed under a high concentration of anesthesia (Figures S3H–S3I). This suggests that the degree of neuronal intracellular ATP response could depend on the dynamics of cortical extracellular serotonin released by photostimulated raphe serotonergic neurons, which probably relies on basal serotonergic activities in a state-dependent manner.^22^^,^^23^ In addition, the degree of cortical neuronal ATP response could be influenced by baseline ATP levels fluctuating with sleep-wake states.^12^

Because the most reliable increase in cortical extracellular serotonin levels and neuronal ATP responses was induced by serotonergic photostimulation during the non-REM sleep state of mice, we adopted a protocol of raphe serotonergic photostimulation during the non-REM sleep state in our subsequent experiments. After we checked the equivalent increase in cortical neuronal ATP levels by serotonergic photostimulations with ChR2(C128S) for 10, 20, and 30 s during the non-REM sleep state (Figure S1G), we further adopted the 30-s photostimulation protocol in our following experiments because it was the most optimal to evaluate the evoked fluctuation of fluorescent signals in the cortex without major interference by the stimulation light. Furthermore, serotonergic photostimulation for 30 s was consistent with the duration of physiological serotonergic activity at wake onset from non-REM sleep in mice.^4^ When we compared the fluctuations in cortical neuronal ATP levels during serotonergic activation-induced awakening and spontaneous awakening from the non-REM sleep or REM sleep states; however, the increase in neuronal ATP levels by serotonergic activation-induced awakening exceeded the increase during the spontaneous awakening from the non-REM sleep state whereas they were equivalent during the awakening from the REM sleep state in terms of the peak and its timing (Figures 1F and 1G).

Next, we conducted pharmacological experiments to confirm whether the dynamics of cortical neuronal ATP levels under raphe serotonergic photostimulation were directly evoked by serotonin released into the cortex. We observed that treatment with fluoxetine, a selective serotonin reuptake inhibitor (SSRI), increased the peak of the neuronal ATP signal response to serotonergic photostimulation (Figures 1H and S4A). We next performed the topical administration of antagonists of serotonin receptors, based on previous reports that cortical pyramidal neurons express serotonin 5-HT_1A_, 5-HT_2A_, 5-HT_2B_, and 5-HT_2c_ receptor subtypes among 14 classes of serotonin receptors.^24^^,^^25^ We observed that the cortical neuronal ATP signal response to serotonergic activation was attenuated by the 5-HT_2A_, 5-HT_2B_, and 5-HT_2c_ receptor antagonists, that is, MDL100907, SB204741, and SB242084, respectively (Figures 1I and S4B–S4F). These results indicate that the increase in cortical neuronal intracellular ATP levels under raphe serotonergic neuronal activation is directly evoked, at least in part, by the release of serotonin.

Nevertheless, we further examined the possibility that on serotonergic activation-induced awakening, other wake-promoting neurons might be activated and simultaneously modulate brain metabolic activities to impact cortical neuronal intracellular ATP dynamics.^26^ We observed that selective lesions of the central noradrenergic system induced by DSP-4 treatment did not affect the cortical neuronal ATP signal response to serotonergic photostimulation (Figures 1J and S4G). In contrast, topical administration of the muscarinic and nicotinic cholinergic receptor antagonists, scopolamine (Sco) and mecamylamine/methyllycaconitine (M/M), respectively, attenuated the cortical neuronal ATP signal response to serotonergic activation (Figures1J and S4H–S4I). These suggest that the central cholinergic system could be involved in a regulatory mechanism of brain energy metabolism to increase the cortical neuronal intracellular ATP levels under the control of the central serotonergic system, whereas the central noradrenergic system could regulate brain energy metabolism independent of the serotonergic system.^27^

### Neuronal lactate uptake is involved in serotonergic activation-induced neuronal intracellular ATP enhancement

**Figure 2:**
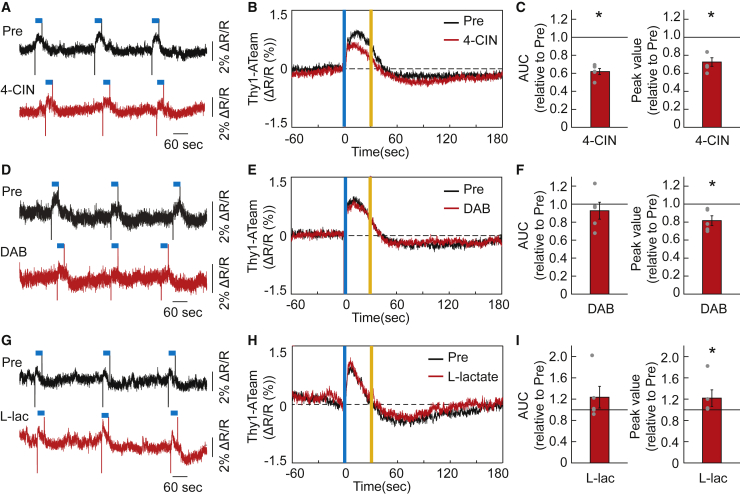
Lactate mediates the increase in intracellular ATP levels in cortical neurons by activating raphe serotonergic neurons (A) Representative trace of diminished cortical neuronal intracellular ATP (Thy1-ATeam) signal responses to serotonergic photostimulation by topical administration of 4-CIN in comparison with Pre (before drug administration) in the same mouse. The blue line above the data represents the timing of serotonergic photostimulation for 30 s. (B) Effect of 4-CIN on cortical neuronal intracellular ATP dynamics under serotonergic photostimulation is shown as averaged traces of Thy1-ATeam signals before (Pre) and after the administration (n = 5 sessions from 5 mice). Vertical blue and yellow lines indicate the 1-s illumination of each light color. (C) Alteration of AUC and peak value of Thy1-ATeam signal responses to serotonergic activation by 4-CIN administration. ∗p< 0.05 versus Pre (before drug administration); two-sided Wilcoxon signed-rank test (n = 5 sessions from 5 mice). (D) Representative trace of blunted Thy1-ATeam signal responses to serotonergic photostimulation by topical administration of DAB in comparison with Pre (before drug administration) in the same mouse. (E) Effect of DAB on neuronal intracellular ATP dynamics under serotonergic photostimulation is shown as averaged traces of Thy1-ATeam signals (n = 5 sessions from 5 mice). (F) Alteration of AUC and peak value of Thy1-ATeam signal responses to serotonergic activation by treatment with DAB for the data in (D and E). ∗p< 0.05 versus Pre; two-sided Wilcoxon signed-rank test (n = 5 sessions from 5 mice). (G) Representative trace of sharpened Thy1-ATeam signal responses to serotonergic photostimulation by topical administration of L-lactate in comparison with Pre (before drug administration) in the same mouse. (H) Effect of L-lactate on neuronal intracellular ATP dynamics under serotonergic photostimulation. is shown as averaged traces of Thy1-ATeam signals (n = 5 sessions from 5 mice). (I) Alteration of AUC and peak value of Thy1-ATeam signal responses to serotonergic activation by treatment with L-lactate for the data in (G and H). ∗p< 0.05 versus Pre; two-sided Wilcoxon signed-rank test (n = 5 sessions from 5 mice). Data are expressed as the mean ± SEM. See also Figure S4.

Because the neuronal ATP signal response to serotonergic activation was attenuated by antagonists of 5-HT_2B_ and 5-HT_2C_ receptor subtypes (Figures 1I, S4E, and S4F), which have been reported to be involved in the regulation of glycogenolysis in astrocytes as well as expressed in neurons,^15^^,^^16^^,^^28^ we next hypothesized that some of released serotonin acts on cortical astrocytes and modulates their metabolic activities to support neuronal energy synthesis. The serotonergic system can regulate the ANLS, which consists of astrocytic lactate production by glycolysis and glycogenolysis, and its supply to neighboring neurons, which could contribute to prompt ATP production in cortical neurons. To test this hypothesis, we topically injected α-cyano-4-hydroxycinnamate (4-CIN), a blocker of monocarboxylate transporter 2 (MCT2) in neurons, and 1,4-dideoxy-1,4-imino-D-arabinitol (DAB), an inhibitor of glycogen phosphorylation in astrocytes (Figure 2).^29^^,^^30^ We observed that the 4-CIN administration attenuated the neuronal ATP signal response to serotonergic activation (Figures 2A–2C and S4J). And the DAB administration slightly but significantly blunted the peak value of neuronal ATP signal response to serotonergic activation, although it did not affect the total amount of increase in neuronal ATP signal response (Figures 2D–2F and S4K). Furthermore, the peak value of the ATP signal response to serotonergic activation was enhanced by the topical administration of L-lactate, whereas it also did not affect the total amount of increase in neuronal ATP signal response (Figures 2G–2I and S4L). These results indicate that serotonergic activation-induced increase in cortical neuronal ATP levels at least partially requires neuronal uptake of extracellular lactate, probably derived from glycogen storage in astrocytes.

**Figure 3:**
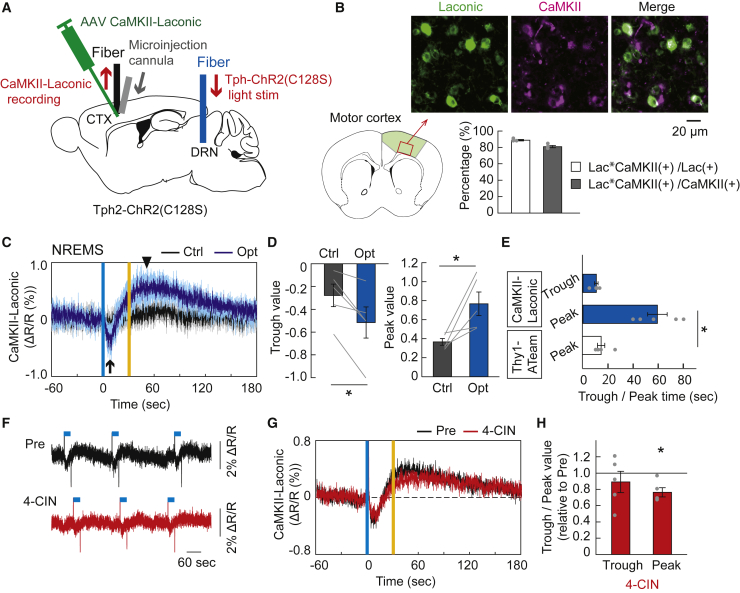
Fluctuation of intracellular lactate levels of cortical neurons by activating raphe serotonergic neurons (A) Schematic illustration of the fiber photometric recording of intracellular lactate levels in cortical pyramidal neurons under the raphe serotonergic photostimulation in Tph2-ChR2(C128S) mice injected with AAV-CaMKII-Laconic. CTX, cortex; DRN, dorsal raphe nucleus. (B) Top, Laconic expression in CaMKII-positive pyramidal neurons in layer 5 of the motor cortex. Scale bar, 20 μm. Bottom, Percentage of neurons labeled with Laconic and CaMKII (1789 Laconic-positive cells and 1950 CaMKII-positive cells, in 4 mice). Lac, Laconic. (C) Traces of CaMKII-Laconic signals in the cortex under serotonergic photostimulation (Opt) and control light illuminations (Ctrl) during the NREM sleep state. Signal traces represent mean ± SEM (n = 5 sessions from 5 mice). Note that CaMKII-Laconic signal showed an initial trough (black arrow) followed by an increase (black arrowhead) under serotonergic photostimulation. Vertical blue and yellow lines indicate 1-s illumination of each light color. In the control condition, yellow light illumination was used in place of blue light. (D) Initial trough and following peak value of CaMKII-Laconic signal responses to serotonergic photostimulation. ∗p< 0.05 versus Control; two-sided Wilcoxon signed-rank test (n = 5 sessions from 5 mice). (E) Initial trough and following peak time of CaMKII-Laconic signals, compared with the peak time of Thy1-ATeam signals. ∗p< 0.05 versus peak time of Thy1-ATeam; Kruskal–Wallis test with *post hoc* Steel test (n = 5 sessions from 5 mice for CaMKII-Laconic and Thy1-ATeam, respectively). (F) Representative trace of altered CaMKII-Laconic signal responses to serotonergic photostimulation by topical administration of 4-CIN in comparison with Pre (before drug administration) in the same mouse. The blue line above the data represents the timing of serotonergic photostimulation for 30 s. (G) Comparison of averaged CaMKII-Laconic signal responses to serotonergic photostimulation between before and after the 4-CIN treatment (n = 5 sessions from 5 mice). (H) The effect of 4-CIN on trough and peak value of CaMKII-Laconic signal responses to serotonergic photostimulation. *p*< 0.05 versus Pre; two-sided Wilcoxon signed-rank test (n = 5 sessions from 5 mice). Data are expressed as mean ± SEM. See also Figure S5.

Next, we monitored the dynamics of intracellular lactate levels in cortical pyramidal neurons under serotonergic activation using a genetically encoded lactate sensor, Laconic.^31^ We injected an AAV expressing Laconic with pyramidal neuron-specific CaMKII promoter (CaMKII-Laconic) into the cortex of Tph2-ChR2(C128S) mice and measured the CaMKII-Laconic fluorescent signals using fiber photometry (Figure 3A). We quantified the specificity and efficiency of Laconic positive pyramidal neurons in the cortex and found that 88.7 ± 0.9% of Laconic expressing neurons co-expressed CaMKII, and 80.9 ± 1.4% of CaMKII-expressing neurons co-expressed Laconic (Figure 3B). The intracellular lactate levels in cortical pyramidal neurons initially decreased, followed by an increase under serotonergic photostimulation during the non-REM sleep state (Figures 3C–3E). This serotonergic activation-induced signal fluctuation was not seen in the ratio of CaMKII-CFP and CaMKII-mVenus fluorescence intensity as a control (Figure S5A). Under serotonergic photostimulation during the wake state, the temporal decrease in neuronal lactate levels diminished (Figures S5B–S5D). The initial decrease of neuronal lactate levels to a minimum was the same time as the intracellular ATP levels peaked, followed by the neuronal lactate increased (Figure 3E). From these findings, it is assumed that the transient decrease in neuronal intracellular lactate levels under serotonergic activation could reflect accelerated lactate consumption for ATP synthesis over lactate synthesis and uptake from extracellular lactate in neurons, whereas the subsequent increase in neuronal intracellular lactate levels could reflect the synthesis and uptake of lactate in neurons exceeding the utilization of lactate. Next, to clarify the effect of neuronal uptake of extracellular lactate on the intracellular lactate dynamics under serotonergic activation, we topically injected 4-CIN, a blocker of MCT2 in neurons. We observed that the administration of 4-CIN attenuated the peak of neuronal intracellular lactate levels increase reached after serotonergic activation, whereas no significant changes in the dynamics of neuronal intracellular lactate levels during serotonergic activation was detected (Figures 3F–3H, S5E, and S5F). The attenuation of the serotonergic activation-induced late increase in neuronal intracellular lactate levels by the 4-CIN treatment could directly reflect the failure of lactate uptake in neurons. On the other hand, the transient decrease in lactate signals during serotonergic activation, which could reflect both neuronal uptake of extracellular lactate and consumption of intracellular lactate for prompt ATP synthesis, was seemingly unaffected by the 4-CIN treatment. This suggests that 4-CIN treatment could induce a decrease in basal intracellular lactate concentration rather than its relative dynamics during serotonergic photostimulation. Regretfully, however, it is technically difficult to demonstrate the reduction of neuronal intracellular lactate concentration *in vivo* before and after 4-CIN administration because of the fluorescence fading of the Laconic sensor.

### Serotonergic activation increases the extracellular lactate level

**Figure 4:**
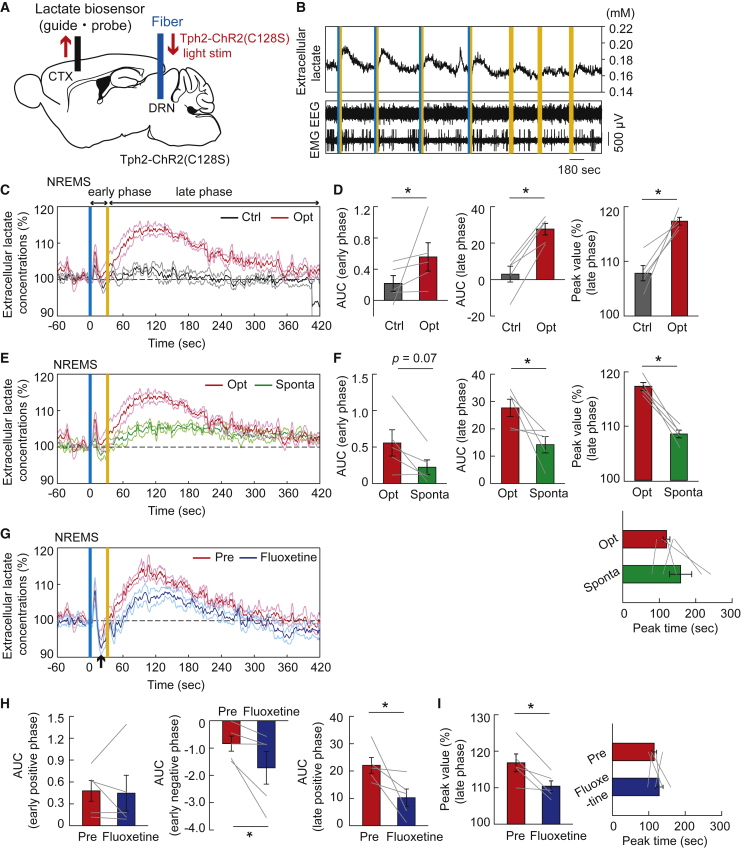
Cortical extracellular lactate concentration was raised by the activation of raphe serotonergic neurons (A) Schematic illustration of recording of cortical extracellular lactate concentrations under the raphe serotonergic photostimulation in Tph2-ChR2(C128S) mice. CTX, cortex; DRN, dorsal raphe nucleus. (B) Representative traces of cortical extracellular lactate concentrations and EEG and EMG signals under serotonergic photostimulation and control light illuminations. Vertical blue and yellow lines indicate 1-s illumination of each light color. In the control condition, yellow light illumination was used in place of blue light. (C) Traces of extracellular lactate signals in the cortex under serotonergic photostimulation (opt) and control light illuminations (Ctrl). Signal traces represent mean ± SEM (n = 5 sessions from 5 mice). We refer to the early phase during serotonergic photostimulation for 30 s and the late phase after the photostimulation, respectively. (D) AUC in the early phase (left) and late phase (middle), and peak value of late phase (right) of the extracellular lactate response to serotonergic photostimulation for the data in (C). ∗p< 0.05 versus Control; two-sided Wilcoxon signed-rank test (0–30 s for early phase and 30–400 s for late phase; n = 5 sessions from 5 mice). (E) Traces of averaged cortical extracellular lactate dynamics under serotonergic photostimulation-induced awakening (Opt) and spontaneous awakening (Sponta) from NREM sleep (n = 5 sessions from 5 mice). (F) Comparison of AUC in early phase (left) and late phase (middle), and peak value (right) and its time (below right) of the extracellular lactate dynamics between serotonergic photostimulation-induced and spontaneous awakening for the data in (E). ∗p< 0.05 versus Sponta; two-sided Wilcoxon signed-rank test (n = 5 sessions from 5 mice). (G) Comparison of averaged cortical extracellular lactate dynamics responding to serotonergic photostimulations between before (Pre) and after the fluoxetine treatment. Note that a temporary dip (black arrow) appeared immediately after the peak in the early phase under the fluoxetine treatment, so they were subsequently analyzed separately as early positive and negative phases. (H) The effect of fluoxetine on the AUC of the early positive phase (left), early negative phase (middle), and late positive phase (right) of the extracellular lactate response between before (Pre) and after the treatment for the data in (G). ∗p< 0.05 versus Pre; two-sided Wilcoxon signed-rank test (0–30 s for early phase and 30–400 s for late phase; n = 5 sessions from 5 mice). (I) The effect of fluoxetine on peak value and the peak time of the extracellular lactate response between before and after the treatment for the data in (G). ∗p< 0.05 versus Pre; two-sided Wilcoxon signed-rank test (n = 5 sessions from 5 mice). All traces and data of extracellular lactate signals represent as mean ± SEM.

Because raphe serotonergic activation could promote lactate uptake in cortical neurons, we next investigated the cortical extracellular lactate concentration dynamics under serotonergic photostimulation using biosensor electrodes (Figure 4A). Serotonergic photostimulation for 30 s during the non-REM sleep led to an increase in cortical extracellular lactate concentration, inducing the awakening of animals (Figures 4B and 4C). Serotonergic photostimulation caused a bimodal increase in extracellular lactate concentration, with a fast increase during serotonergic stimulation (referred to as the early phase) and a slow increase after the stimulation (late phase) (Figures 4C and 4D). When we compared the dynamics of cortical extracellular lactate levels during serotonergic activation-induced awakening and spontaneous awakening lasting more than 30 s from non-REM sleep,^10^^,^^32^ serotonergic activation-induced increase in cortical extracellular lactate levels exceeded that at the spontaneous awakening of mice, especially in the late phase, whereas the peak timing was not different (Figures 4E and 4F). Of interest, treatment with fluoxetine, a selective serotonin reuptake inhibitor (SSRI) that potentiates the effects of serotonergic photostimulation, significantly attenuated the serotonergic activation-induced increase in cortical extracellular lactate in the late phase and brought a transient decrease in lactate levels between the initial small and later large increases, whereas it did not affect the initial increase in lactate levels in the early phase (Figures 4G–4I). This suggests that fluoxetine might promote serotonergic activation-evoked lactate uptake of cortical neurons, especially in the late phase more than it could promote lactate production and release to the extracellular space from presumable astrocytes.^33^^,^^34^ It should be noted, however, that our prediction was not supported experimentally, because we observed that the attenuation of serotonergic activation-induced increase in cortical extracellular lactate concentration by fluoxetine treatment was not restored by topical administration of 4-CIN, a blocker of MCT2 in neurons. Further hypotheses are needed for mechanisms of the effects of SSRI on extracellular lactate dynamics.

### Optogenetic activation of serotonergic neurons induces cortical astrocytic cAMP and Ca^2+^ surges

**Figure 5:**
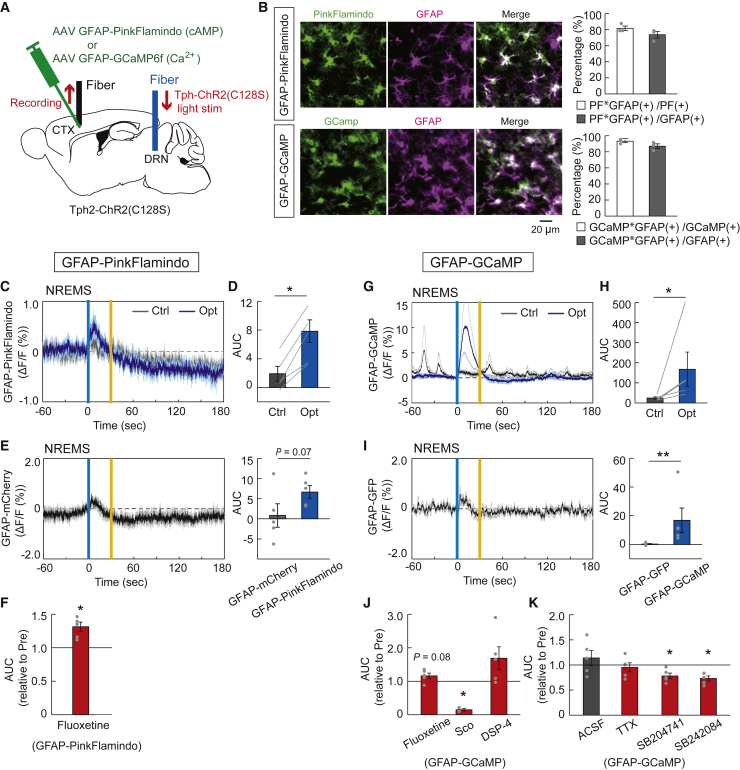
Response of intracellular cAMP and Ca^2+^ signals in cortical astrocytes to the activation of raphe serotonergic neurons (A) Schematic illustration of the fiber photometric recording of astrocytic intracellular cAMP and Ca^2+^ signals in the cortex under the raphe serotonergic photostimulation in Tph2-ChR2(C128S) mice injected with AAV-GFAP-PinkFlamindo and AAV-GFAP-GCaMP6f, respectively. CTX, cortex; DRN, dorsal raphe nucleus. (B) Left, PinkFlamindo (top) and GCamp (bottom) expression in GFAP-positive astrocytes in layer 5 of the motor cortex. Scale bars, 20 μm. Right, for GFAP-PinkFlamindo (top), the percentage of cells labeled with PinkFlamindo and GFAP (2958 PinkFlamindo-positive cells and 3256 GFAP-positive cells, in three mice). For GFAP-GcaMP (bottom), the percentage of cells labeled with GCamp and GFAP (1394 GCamp-positive cells and 1507 GFAP-positive cells, in three mice). (C) Traces of GFAP-PinkFlamindo signals in the cortex under serotonergic photostimulation (Opt) and control light illumination (Ctrl). Signal traces represent mean ± SEM (n = 5 sessions from 5 mice). Vertical blue and yellow lines indicate 1-s illumination of each light color. In the control condition, yellow light illumination was used in place of blue light. (D) AUC of GFAP-PinkFlamindo signal responses to the raphe serotonergic photostimulation. ∗p< 0.05 versus Control; two-sided Wilcoxon signed-rank test (n = 5 sessions from 5 mice). (E) Traces of averaged control GFAP-mCherry fluorescence intensity fluctuations (left) and comparison between GFAP-mCherry and GFAP-PinkFlamindo fluorescent signals (right). Mann–Whitney test (n = 5 sessions from 4 mice for GFAP-mCherry and n = 5 sessions from 5 mice for GFAP-PinkFlamindo). (F) The effect of fluoxetine on AUC of GFAP-PinkFlamindo signal responses to serotonergic photostimulation. ∗p< 0.05 versus Pre (before drug administration); two-sided Wilcoxon signed-rank test (n = 5 sessions from 5 mice). (G) Traces of averaged GFAP-GCaMP signals in the cortex under serotonergic photostimulation (Opt) and control light illumination (Ctrl) (n = 5 sessions from 5 mice). (H) AUC of GFAP-GCaMP signal responses to serotonergic photostimulation. ∗p< 0.05 versus Control; two-sided Wilcoxon signed-rank test (n = 5 sessions from 5 mice). (I) Traces of averaged control GFAP-GFP fluorescence intensity fluctuations (left) and comparison between GFAP-GFP and GFAP-GCaMP fluorescent signals (right). ∗∗p< 0.01 versus GFAP-GFP; Mann–Whitney test (n = 5 sessions from 3 mice for GFAP-GFP and n = 5 sessions from 5 mice for GFAP-GCaMP). (J) The effect of fluoxetine, scopolamine, and DSP-4 on AUC of GFAP-GCaMP signal responses to serotonergic photostimulation. ∗p< 0.05 versus Pre (before the treatment); two-sided Wilcoxon signed-rank test (n = 5 sessions from 5 mice). (K) The effect of ACSF (control), TTX, SB204741, and SB242084 on AUC of GFAP-GCaMP signal responses to serotonergic photostimulation. ∗p< 0.05 versus Pre (before the administration); two-sided Wilcoxon signed-rank test (n = 5 sessions from 5 mice). Data are expressed as mean ± SEM. See also Figures S6 and S7.

Next, we investigated the possibility that raphe serotonergic activation could induce the activation of astrocytic second messengers, cyclic adenosine monophosphate (cAMP), and Ca^2+^ signaling, intricately related to each other to trigger astrocytic glycogenolysis and lactate release^35^^,^^36^ as a cause of the increase in extracellular lactate levels. We injected an AAV expressing PinkFlamindo^37^ or GCaMP6f with an astrocyte-specific GFAP promoter into the cortex of Tph2-ChR2(C128S) mice and measured GFAP-PinkFlamindo or GFAP-GCaMP6f fluorescent signals using fiber photometry (Figure 5A). We confirmed that PinkFlamindo and GCaMP6f are selectively expressed in GFAP-positive astrocytes. For GFAP-PinkFlamindo, 81.6 ± 3.0% of PinkFlamindo-positive cells co-expressed GFAP and 73.6 ± 4.1% of GFAP-positive astrocytes expressed PinkFlamindo. For GFAP-GCaMP6f, 93.1 ± 1.6% of GCaMP6f-positive cells co-expressed GFAP and 87.0 ± 3.0% of GFAP-positive astrocytes expressed GCaMP6f (Figure 5B). Both the cortical astrocytic cAMP and Ca^2+^ signals increased under raphe serotonergic photostimulations for 30 s during the non-REM sleep state in mice (Figures 5C, 5D, 5G, and 5H). The GFAP-PinkFlamindo and GFAP-GCaMP fluorescent signal increases under serotonergic stimulations were greater than the control fluorescent fluctuations of GFAP-mCherry and GFAP-GFP, respectively, although it should be noted that the fluorescent fluctuation of GFAP-PinkFlamindo was relatively close to that of GFAP-mCherry (Figures 5E and 5I). The astrocytic cAMP signal response to serotonergic activation was enhanced by treatment with the SSRI fluoxetine (Figures 5F and S6). The astrocytic Ca^2+^ signal showed an increased peak value of response to serotonergic activation by fluoxetine treatment, although the AUC in astrocytic Ca^2+^ signal response was not significantly altered. In contrast, the astrocytic Ca^2+^ signal response was significantly attenuated by treatment with scopolamine, a muscarinic cholinergic receptor antagonist. The astrocytic Ca^2+^ signal seems to be affected by treatment with DSP-4, a selective neurotoxin for the central noradrenergic system; however, this effect was not statistically significant (Figures 5J, S7A–S7C, and S7H). These findings suggest that the raphe serotonergic activation-induced Ca^2+^ surge in cortical astrocytes could be partly mediated by central cholinergic systems under the control of central serotonergic systems.

Because the serotonergic system modulates the firing activity of cortical pyramidal neurons,^25^^,^^38^ astrocytic Ca^2+^ and cAMP surges might be induced by local neuronal activity via extracellularly released glutamate and high K^+^ concentrations.^36^^,^^39^^,^^40^ However, our results showed that the astrocytic Ca^2+^ signal response to serotonergic activation was not altered by tetrodotoxin (TTX) treatment (Figures 5K and S7E). The astrocytic Ca^2+^ surge was attenuated by treatment with 5-HT_2B_ and 5-HT_2c_ receptor antagonists SB204741 and SB242084, respectively (Figures 5K and S7F–S7H). This suggests that the astrocytic Ca^2+^ surge under serotonergic activation could be directly induced by the released serotonin without mediating local neuronal activities.

### Chemogenetic inhibition of raphe serotonergic neurons attenuates the increase of cortical neuronal intracellular ATP levels in awakening from REM sleep

**Figure 6:**
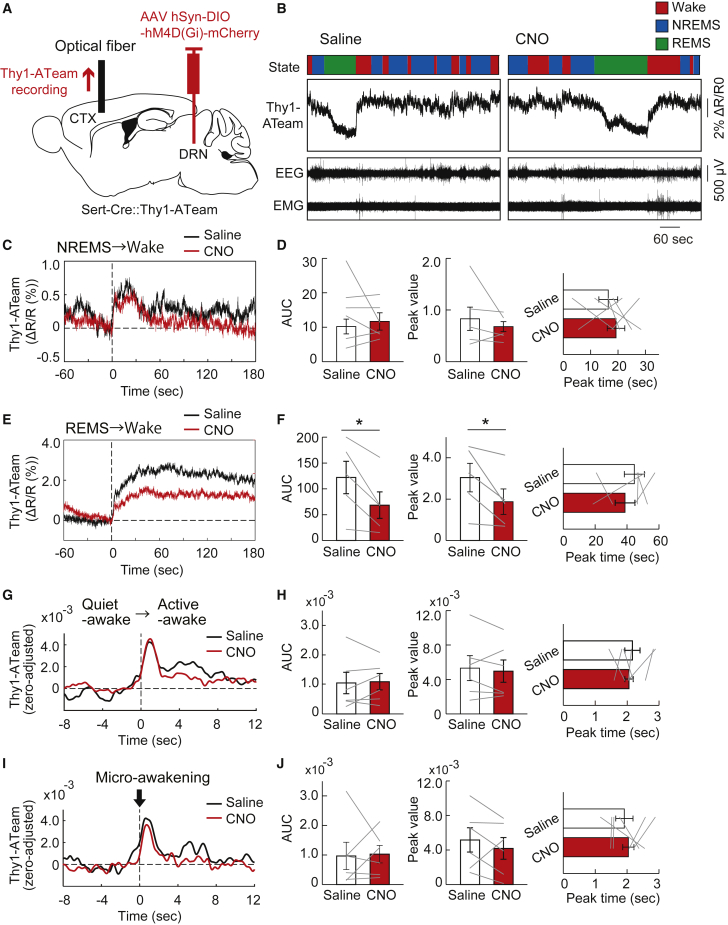
Alteration of state-dependent cortical neuronal intracellular ATP signal dynamics by chemogenic inhibition of dorsal raphe serotonergic neurons (A) Schematic illustration of the fiber photometric recording of intracellular ATP levels in cortical pyramidal neurons in Sert-Cre::Thy1-ATeam mice injected with AAV-hSyn-DIO-hM4D(Gi)-mCherry into the dorsal raphe nucleus. CTX, cortex; DRN, dorsal raphe nucleus. (B) Representative traces of cortical neuronal intracellular ATP signals (Thy1-ATeam), EEG, and EMG signals with vigilance states of mice under saline or CNO administration. (C) Traces of averaged Thy1-ATeam signal in the cortex during the transition from NREM sleep to wake state under saline (control) or CNO administration (n = 6 sessions from 4 mice). (D) AUC (left), peak value (middle), and peak timing (right) of the Thy1-ATeam signal immediately after the transition from NREM sleep to wake state under saline or CNO administration. Two-sided Wilcoxon signed-rank test (n = 6 sessions from 4 mice). (E) Traces of averaged Thy1-ATeam signal during the transition from REM sleep to wake state under saline or CNO administration (n = 5 sessions from 3 mice). (F) AUC (left), peak value (middle), and peak timing (right) of the Thy1-ATeam signal immediately after the transition from REM sleep to wake state under saline or CNO administration. ∗p< 0.05 versus saline; two-sided Wilcoxon signed-rank test (n = 5 sessions from 3 mice). (G) Traces of averaged Thy1-ATeam signal during the transition from the quiet-awake to active-awake substate during the wake state under saline or CNO administration (n = 6 sessions from 4 mice). (H) AUC (left), peak value (middle), and peak timing (right) of the Thy1-ATeam signal immediately after the transition from the quiet-awake to active-awake substate under saline or CNO administration. Two-sided Wilcoxon signed-rank test (n = 6 sessions from 4 mice). (G) Traces of averaged Thy1-ATeam signal at the micro-awakening events during the NREM sleep under saline or CNO administration (n = 6 sessions from 4 mice). (J) AUC (left), peak value (middle), and peak timing (right) of the Thy1-ATeam signal at the micro-awakening events during the NREM sleep under saline or CNO administration. Two-sided Wilcoxon signed-rank test (n = 6 sessions from 4 mice). Bar graphs show mean ± SEM.

Finally, we examined the role of the central serotonergic system in controlling cortical neuronal intracellular ATP dynamics across the vigilance state of the animals. We used inhibitory designer receptors exclusively activated by designer drugs (DREADD) to selectively inhibit the neuronal activities in the dorsal raphe serotonergic neurons. We injected an AAV expressing DIO-hM4D(Gi)-mCherry with hSyn promoter in Sert-Cre::Thy1-ATeam double-transgenic mice (Figure 6A). We administered saline or clozapine-N-oxide (CNO) intraperitoneally in mice expressing hM4Di in the dorsal raphe serotonergic neurons and recorded cortical Thy1-ATeam signal and EEG/EMGs for several subsequent hours. Compared with saline administration, CNO significantly attenuated the increase in cortical neuronal intracellular ATP levels during the transition from REM sleep to wake state, whereas it did not affect those during the transition from non-REM sleep to wake state (Figures 6B–6F). When we evaluated the effect of raphe serotonergic neuronal activities on the dynamics of cortical neuronal ATP levels between sub-states within the sleep/wake states,^12^ CNO administration did not change any dynamics of cortical neuronal intracellular ATP levels during the transition from quiet-awake to active-awake substates within the wake state or micro-awakening events during the non-REM sleep state (Figures 6G–6J). These findings suggest that the dorsal raphe serotonergic neurons are involved in the regulation of state-dependent dynamics of cortical neuronal intracellular ATP levels and partly contribute to the enhancement of cortical neuronal intracellular ATP levels associated with an increase in arousal level in animals.

## Discussion

The present study demonstrates that raphe serotonergic neuronal activation induces an increase in cortical neuronal intracellular ATP levels, partly mediated by the neuronal uptake of extracellular lactate supplied from astrocytes with an increase in arousal level in mice. Serotonergic activation also induces a surge in cortical astrocytic intracellular cAMP and Ca^2+^ signals and an increase in cortical extracellular lactate concentration, suggesting that the serotonergic system facilitates ANLS. Furthermore, selective inhibition of raphe serotonergic neurons partly attenuated the increase in cortical neuronal intracellular ATP levels as arousal increased in mice. These findings suggest that serotonergic functions for regulating cerebral energy metabolism could partly contribute to the state-dependent increase in cortical neuronal energy levels.

**Figure 7:**
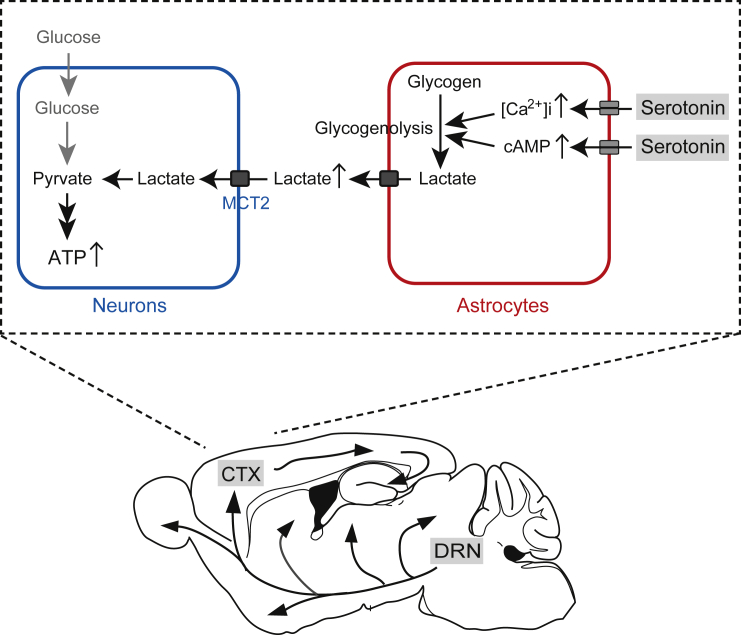
Serotonergic control of ANLS and neuronal energy levels Serotonin increases astrocytic cAMP and Ca^2+^ signals, triggering glycogenolysis for lactate production and its extracellular release. In turn, neurons increase intracellular ATP levels partly via lactate uptake from the extracellular space. CTX, cortex; DRN, dorsal raphe nucleus.

The serotonergic function in regulating brain energy metabolism has not received much attention for a long time, except for its vasoconstrictive function.^41^ The serotonergic vasoconstrictive function is expected to result in a decrease in the metabolic rate of glucose consumption, which mainly reflects glycolysis for ATP synthesis in neurons and astrocytes.^42^ In combination with serotonin-induced vasoconstriction, our new insight on the serotonergic function of ANLS facilitation suggests that intense serotonergic neuronal activation could switch the cortical neuronal energy substrates from glucose to lactate derived from astrocytes, which could result in an overall increase in neuronal intracellular ATP levels (Figure 7). Otherwise, it is possible that serotonergic neuronal activation could promote neuronal/astrocytic glycolysis, dissociated from hemodynamic changes, by increasing the permeability of the blood-brain barrier via undetermined mechanisms in astrocytes and promoting the cellular uptake of glucose.^41^ These multidimensional regulations of cerebral energy metabolism by the central serotonergic system could explain the imperfect effect on the increase of cortical neuronal intracellular ATP levels with arousal increase in mice with inhibited serotonergic neurons (Figure 6).

We concluded that serotonergic activation promotes ANLS based on the finding of an increase in extracellular lactate concentrations by serotonergic neuronal activation (Figure 4), according to the knowledge that the increase in extracellular lactate concentration is mainly because of release from astrocytes.^33^^,^^34^ Furthermore, astrocytic cAMP and Ca^2+^ signaling, evoked by serotonergic neuronal activation (Figure 5), are known to trigger lactate production via glycolysis and glycogenolysis in astrocytes and their extracellular release.^35^^,^^36^ Several factors, such as noradrenaline, adenosine, and high extracellular K^+^ levels, have been reported to promote astrocytic lactate production directly or through cAMP and Ca^2+^ signaling.^18^^,^^36^ Our data are consistent with previous reports that the activation of serotonin 5-HT_2B_ or 5-HT_2C_ receptor subtypes induces glycogenolysis and an increase in intracellular Ca^2+^ concentration, as well as inducing other signaling cascades such as ERK phosphorylation in astrocytes.^15^^,^^16^^,^^43^ On the other hand, our finding of the serotonin-induced astrocytic cAMP surge has not been previously reported, although astrocytic cAMP surge is expected to be induced via the activation of other serotonin receptor subtypes.^44^ Furthermore, importantly, it is well known that astrocytic glycogenolysis and ANLS are required for neuronal activities and long-term memory.^45^^,^^46^^,^^47^^,^^48^ In addition to extracellular lactate functioning as a neuro/gliotransmitter as well as an energy intermediate, neuronal intracellular ATP is not only a ready-to-use cellular energy substance but is also expected to function in modulating neuronal firing activities via ATP-sensitive potassium channels.^18^^,^^49^^,^^50^^,^^51^ Hence, the central serotonergic system is expected to indirectly control neuronal activities and consequent brain functions, such as memory, via the regulation of ANLS and neuronal intracellular ATP levels, as well as directly control neuronal firing activities in each widespread projecting brain region.^17^^,^^52^

In the present study, we demonstrated that selective inhibition of raphe serotonergic neuronal activity attenuates the increase in cortical neuronal intracellular ATP levels with the awakening of mice from REM sleep (Figure 6). This reduction in the state-dependent increase in cortical neuronal ATP levels by serotonergic inhibition could not only cause a shortage of cellular energy for normal neuronal activities/functions but also might affect neuronal firing activity by modulation via ATP-sensitive potassium channels.^18^^,^^49^^,^^50^^,^^51^ In addition, in the background of impaired state-dependent adaptation of neuronal ATP levels because of serotonergic inhibition, unachieved astrocytic activation and ANLS promotion in awaking animals could also be expected. In turn, the impaired adaptation of neuronal ATP levels and the resulting abnormal neuronal activities in a wide range of brain areas with abnormal astrocytic activities and dynamics of extracellular lactate levels under the dysregulation of the serotonergic system are likely to cause abnormal behavior in animals. It has been elucidated that astrocytic dysfunction is involved in conditions, such as depression-like behavior and memory impairment, which are known to be symptoms of stress and depression associated with the aberrant central serotonergic system.^46^^,^^53^^,^^54^^,^^55^ Furthermore, the dysregulation of neuronal/astrocytic energy metabolism by the serotonergic system is expected to be involved in symptoms such as depression-like behavior and memory impairment. Further studies are needed to verify this.

Because we conducted *in vivo* optogenetic activation of raphe serotonergic neurons to elucidate the effect on cerebral energy metabolism, this was always accompanied by the arousal level in mice indicated by EEG change, even under general anesthesia (Figures 1 and S2). Hence, our findings on serotonergic activation-induced cerebral metabolic changes could be influenced by other wake-promoting neuronal systems, which are possibly activated during serotonergic activation-induced awakening in mice. Using pharmacological experiments to test this possibility, we found that the central cholinergic system, one of the wake-promoting neurons for those localized in the basal forebrain (BF),^56^^,^^57^ is facilitatively involved in serotonergic activation-induced astrocytic Ca^2+^ surge and increase in cortical neuronal ATP levels (Figures 1 and 5). BF cholinergic neurons have been reported to modulate cerebral astrocytic Ca^2+^ signaling, thereby contributing to synaptic plasticity and neurovascular coupling.^58^^,^^59^ However, little is known about the involvement of the BF cholinergic system in ANLS regulation. Our results suggest that the central cholinergic system could play a role in the regulation of brain energy metabolism, including ANLS, under the control of serotonergic systems. Because no direct projections of central serotonergic neurons to the BF cholinergic neurons have been reported,^60^ further studies are needed to elucidate the anatomical and functional connections between the BF cholinergic systems and central serotonergic systems for the regulation of brain energy metabolism. On the other hand, the central noradrenergic neurons, one of the other wake-promoting systems in animals, have been reported to have multiple functions in the regulation of brain energy metabolism, including the astrocytic Ca^2+^ and cAMP surge and resulting ANLS facilitation.^27^^,^^61^^,^^62^^,^^63^ From our findings in noradrenergic lesion experiments (Figures 1 and 5), we conclude that the central serotonergic and noradrenergic systems could be independently involved in the regulation of brain energy metabolism.

In this study, to elucidate the dynamics of neuronal/astrocytic intracellular metabolic and signaling molecules under the regulation of raphe serotonergic neurons, we employed *in vivo* fluorescence measurement of each probe using fiber photometry. As the control measurement, we evaluated the fluorescent fluctuations of fluorescent proteins in the wavelength bands matching each fluorescent probe expressed under the same promoter to check the effect of the body movement of animals and hemodynamics on the fluorescent signals. We found that all probes showed fluorescent signals significantly different from the corresponding controls except the GFAP-PinkFlamindo, a probe for detecting the cAMP signal in astrocytes. We found it somewhat difficult to detect the fluctuation of the fluorescent signal of GFAP-PinkFlamindo with high confidence compared with that of the control (Figure 5). This problem is expected to be solved in the future with the development of a specific fluorescent probe with a much higher signal/noise ratio. In addition, it could be useful to observe the probe fluorescence using microscopy *in vivo* to minimize noise derived from autofluorescence in the tissue, blood flow, and body movement of animals, although it becomes difficult to execute microinjection of drugs into the brain observation field to clarify its effect on the metabolic signals.

Another technical limitation is the difficulty in calibrating the fluorescent signal of ATP and other metabolites and signaling molecules obtained by fiber photometry to absolute cytosolic concentrations *in vivo*.^12^ Nonetheless, because the serotonergic photostimulation-evoked or spontaneous fluctuation of cortical Thy1-ATeam signals are comparable with the cortical Thy1-ATeam signal responses to local electrical stimulations in a stimulus frequency-dependent manner,^12^ that could be within the dynamic range of the ATeam1.03^YEMK^ probe with its dissociation constant (Kd) of 1.2 mM.^14^ Furthermore, given that the neuronal ATP concentration has been estimated to be approximately 2 mM *ex vivo*,^64^^,^^65^^,^^66^ the serotonergic photostimulation-evoked fluctuations of neuronal ATP concentrations are assumed to be in the range of a few hundred micromoles or less.

The central serotonergic system could be involved in multiple psychiatric disorders, such as affective and anxiety disorders.^67^^,^^68^ Our finding on the metabolic regulatory function of the central serotonergic system suggests that patients with affective and anxiety disorders involving alterations of the central serotonergic system could have underlying pathological alterations in brain energy metabolism and astrocytic activities. In turn, in these patients, it is assumed that aberrant brain energy dynamics could cause pathological neuronal activities and brain functions, as well as a direct effect on neuronal activity by altering the serotonergic system. Our elucidation of the serotonergic function in regulating brain energy metabolism is expected to provide new insights into the pathogenesis of these serotonin-related psychiatric disorders.

### Data availability

Source data used to generate the graphs in the figures are provided in the supplementary figures. All data generated or analyzed during this study are available from the corresponding author on reasonable request.

### Limitations of the study

In the present study, we investigated the regulation of brain energy metabolism by the central serotonergic system with manipulation of serotonergic neuronal activity and measurement of molecular dynamics of brain energy metabolism *in vivo*. However, although the fluorescent probe-based measurement we employed has superior cell type and time specificities, the probe only revealed the dynamics of specific metabolic molecules for which it was developed. Other methodology, such as mass spectrometry, would provide complementary information for comprehensive elucidation of brain energy metabolism regulated by the serotonergic system, although it has neither cell type- nor time-specificity.

In addition, to elucidate the effects of neuronal intracellular ATP levels and supporting ANLS under the central serotonergic regulation on animal behavior, further experiments are required that manipulate these brain energy metabolic activities and molecules rigorously.

## STAR★Methods

### Key resources table

**Table:** 

REAGENT or RESOURCE	SOURCE	IDENTIFIER
**Antibodies**		

Goat polyclonal anti-GFP	Rockland	Cat# 600-101-215; RRID: AB_218182
Mouse monoclonal anti-CaMKII	Abcam	Cat# ab22609; RRID: AB_447192
Rabbit polyclonal anti-GFAP	Sigma-Aldrich	Cat# G9269; RRID: AB_477035
Mouse monoclonal anti-GFAP	Sigma-Aldrich	Cat# G3893; RRID: AB_477010
Rabbit polyclonal anti-DsRed	Clontech	Cat# 632496; RRID: AB_10013483
Donkey anti-goat IgG (H + L) Alexa Fluor 488	Thermo Fisher	Cat# A-11055; RRID: AB_2534102
Goat anti-rabbit IgG (H + L) Alexa Fluor 488	Thermo Fisher	Cat# A11008; RRID: AB_143165
Donkey anti-rabbit IgG (H + L) Alexa Fluor 594	Thermo Fisher	Cat# A21207; RRID: AB_141637
Goat anti-mouse IgG (H + L) Alexa Fluor 594	Thermo Fisher	Cat# A-11005; RRID: AB_2534073

**Bacterial and virus strains**		

AAV9-hSyn-GRAB_5-HT2h_	Wan et al.,^22^ 2021	N/A
AAV9-CaMKII-Laconic	San Martin et al.,^31^ 2013, This paper	Addgene plasmid #44238
AAV9-GFAPp-PinkFlamindo	Harada et al.,^37^ 2017, This paper	Addgene plasmid #102356
AAV5-pZac2.1-gfABC1D-cyto-GCaMP6f	Haustein et al.,^69^ 2014	Addgene viral prep #52925-AAV5
AAV8-hSyn-DIO-hM4D(Gi)-mCherry	Krashes et al.,^70^ 2011	Addgene viral prep #44362-AAV8
AAV9-CaMKII-CFP	This paper	N/A
AAV9-CaMKII-mVenus	This paper	N/A
AAV9-CaMKII-AT1.03RK	Imamura et al.,^14^ 2009, This paper	N/A
AAV9-hSyn-EGFP	Bryan Roth	Addgene viral prep #50465-AAV9.T
AAV-GFAP-GFP	This paper	N/A
AAV-GFAP-mCherry	Perea et al.,^71^ 2014	Addgene viral prep #58909-AAV5

**Chemicals, peptides, and recombinant proteins**		

Tetrodotoxin (TTX)	Wako	CAS: 4368-28-9; Cat# 207–15901
α-Cyano-4-hydroxycinnamic acid (4-CIN)	Sigma-Aldrich	CAS: 28166-41-8; Cat# C2020
1,4-dideoxy-1,4-imino-D-arabinitol hydrochloride (DAB)	Cayman	CAS: 100991-92-2; Cat# 20939
Sodium L-lactate	Sigma-Aldrich	CAS: 867-56-1; Cat# L7022-5G
WAY100635	Tokyo Kasei Kogyo Co.	CAS RN: 1092679-51-0; Cat# W0016
MDL100907	Sigma-Aldrich	CAS: 139290-65-6; Cat# M3324-5MG
SB204741	Sigma-Aldrich	CAS: 152239-46-8; Cat# S0693-10MG
SB242084, Hydrochloride	Cayman	CAS: 1049747-87-6; Cat# 10096
Scopolamine hydrobromide Trihydrate	Tokyo Kasei Kogyo Co.	CAS RN: 6533-68-2; Cat# S0021
Mecamylamine hydrochloride	Cayman	CAS: 826-39-1; Cat# 14602
Methyllycaconitine citrate	Cayman	CAS: 351344-10-0; Cat# 21398
Fluoxetine	Tocris	CAS RN: 56296-78-7; Cat# 0927/10
N-(2-chloroethyl)-N-ethyl-2-bromobenzylamine (DSP-4)	Sigma-Aldrich	CAS No: 40616-75-9; Cat#: 2958/50
Clozapine N-oxide (CNO)	Tocris	CAS No: 34233-69-7; Cat#: 4936

**Experimental models: Organisms/strains**		

Mouse: *Thy1*-ATeam	Trevisiol et. al.,^13^ 2017	RRID: MGI:5882597
Mouse: *Tph2*-tTA	Ohmura et al.,^72^ 2014	RIKEN BRC: RBRC05846
Mouse: tetO-ChR2(C128S)-EYFP	Tanaka et al.,^73^ 2012	RIKEN BRC: RBRC05454
Mouse: SERT-Cre: B6.129(Cg)-Slc6a4^tm1(cre)Xz^/J	Jackson Laboratory	RRID: IMSR_JAX:014554

**Oligonucleotides**		

Primer: *Thy1* promoter Forward: TGCCGGTGTGTTGAGCTA	Trevisiol et. al.,^13^ 2017	N/A
Primer: *Thy1* promoter Reverse: TGG TCCTGTGTTCATTGCTG	Trevisiol et. al.,^13^ 2017	N/A
Primer: *Tph2*-tTA Forward: TCTTCCCAAA GAGCTACTCGACCT	Ohmura et al.,^72^ 2014	N/A
Primer: *Tph2*-tTA Reverse: CGGAGTTGA TCACCTTGGACTTGT	Tanaka et al.,^73^ 2012	N/A
Primer: *tetO-intron* Forward: AGCAGAG CTCGTTTAGTGAACCGT		N/A
Primer: *tetO-intron* Reverse: AAGGCAG GATGATGACCAGGATGT		N/A
Primer: *Cre* recombinase Forward: GGTTCGTTCACTCATGGAAAATAG		N/A
Primer: *Cre* recombinase Reverse: GGTATCTCTGACCAGAGTCATCCT	\	N/A

**Software and algorithms**		

MATLAB	MathWorks	R2017b
Sleepsign	Kissei Comtec	Software ver.3
Sirenia Acquisition Software	Pinnacle Technology	https://www.pinnaclet.com/sirenia.html
Labview	National Instruments	N/A

### Resource availability

#### Lead contact

Further information and requests for resources and reagents should be directed to and will be fulfilled by the lead contact, Akiyo Natsubori (natsubori-ak@igakuken.or.jp).

#### Materials availability

This study did not generate new unique reagents.

### Experimental model and subject details

#### Animals

All animal procedures were conducted in accordance with the National Institutes of Health Guide for the Care and Use of Laboratory Animals and approved by the Animal Research Committee of the Tokyo Metropolitan Institute of Medical Science (approval No. 21–003). All efforts were made to minimize animal suffering and discomfort and to reduce the number of animals used.

Experiments were performed using 3–12-month-old male and female mice. Mice were housed under controlled lighting (12 h light/dark cycle) and temperature (22–24°C) conditions. Food and water were provided *ad libitum*. Experiments were conducted in the latter half of the light phase.

Tph2-ChR2 mice (*Tph2-*tTA::tetO-ChR2(C128S) double-transgenic mice) were obtained by crossing *Tph2*-tTA mice with tetO-ChR2(C128S) mice.^72^^,^^73^ Tph2-ChR2::Thy1-ATeam mice (*Tph2-*tTA::tetO-ChR2(C128S)::*Thy1*-ATeam triple transgenic mice) were obtained by crossing *Tph2*-tTA::tetO-ChR2(C128S) and Thy-ATeam (ATeam1.03^YEMK^) mice.^13^ Sert-Cre::Thy1-ATeam mice (*Sert*-Cre::*Thy1*-ATeam double transgenic mice) were obtained by crossing *Sert*-Cre mice (JAX stock #014554)^74^ and Thy1-ATeam mice. The genetic background of all transgenic mice was C57BL6J and 129 SvEvTac. The following polymerase chain reaction (PCR) primer sets were used for mouse genotyping: Tph-314U (5′-TCTTCCCAAAGAGCTACTCGACCT-3′) and mtTA24L (5′- CGGAGTTGATCACCTTGGACTTGT-3′) for *Tph2*-tTA mice; and tetO-up (5′-AGCAGAGCTCGTTTAGTGAACCGT-3′) and intron-low (5′-AAGGCAGGATGATGACCAGGATGT-3′) for tetO-ChR2(C128S) mice; ThyATPA-S (5′-TCTGAGTGGCAAAGGACCTTAGG-3′) and ThyATPA-AS (5′-CGCTGAACTTGTGGCCGTTTACG-3′) for Thy1-ATeam mice; Cre-F(5′-GGTTCGTTCACTCATGGAAAATAG-3′) and Cre-R (5′-GGTATCTCTGACCAGAGTCATCCT-3′) for *Sert*-Cre mice.

### Method details

#### Virus preparation

The following viruses were acquired from Addgene: AAV5-pZac2.1-gfABC1D-cyto-GCaMP6f (Addgene viral prep # 52925-AAV5, gifted from Baljit Khakh),^69^ AAV9-hSyn-EGFP (Addgene viral prep # 50465-AAV9.T, gifted from Bryan Roth), AAV-GFAP-mCherry (Addgene viral prep # 58909-AAV5, gifted from Edward Boyden),^71^ and AAV8-hSyn-DIO-hM4D(Gi)-mCherry (Addgene viral prep # 44362-AAV8, gifted from Bryan Roth).^70^

The plasmids of Laconic (Addgene plasmid # 44238, gifted from Felipe Barros)^31^ and PinkFlamindo (Addgene plasmid # 102356, gifted from Tetsuya Kitaguchi)^37^ were obtained from Addgene. The plasmid of AT1.03RK (AT1.03^R122K/R126K^)^14^ was provided by Hiromi Imamura.

AAV production was performed using iodioxanol discontinuous gradient according to Zolotukhin et al.^75^ 293T cells were plated onto five plates of 500 cm^2^ (245 mm × 245 mm) culture square dishes in standard cell culture medium (D-MEM, 10% heat-inactivated fetal bovine serum (HI-FBS), 1% penicillin-streptomycin, 2 mM glutamine). At 90% confluency, the transfection was performed with PEI-Max in OPTI-MEM. The 60% of the medium was changed 1 hr before transfection. The transfection mix contained a triple plasmid system (pPack2/9, pHelper, and the viral genome containing plasmid; 51.75 mg of each for one square dish). The medium was changed to 2% HI-FBS containing D-MEM 6 hrs after transfection. Cells were scraped and collected 3 days after transfection and stored at −80°C. Cells in PBS were freeze-thawed between a 37°C water bath and a cooled ethanol bath using dry ice at three cycles and then incubated for 60 min in Benzonase (Merck-Millipore) nuclease in 5 mM MgCl_2_, and the reaction was stopped by EDTA (6.5 mM). After centrifugation at each freeze-thaw cycle, its supernatant was added as the top layer of a centrifuge column (UltraClear Tube (25 × 89 mm); Beckman 344058) filled with an iodixanol (OptiPrep, SEW) gradient (15%, 25%, 40%, and 54% iodixanol layers). After ultracentrifugation for 18 hrsat 28,000 rpmat 10°C, iodixanol layers were extracted from the bottom half of 40% to the top quarter of 54% (about 3.0 mL) with a 19G syringe and concentrated in HEPES-based saline for several cycles using Vivaspin 100K (Sartorius). Samples were finally aliquoted and stored at −80°C. The titer of AAV was estimated by quantitative PCR, and purity was confirmed by SDS-PAGE.

#### Surgical procedure

Stereotaxic surgery was performed under anesthesia with a ketamine-xylazine mixture (100 and 10 mg/kg, i.p.).

For optogenetic manipulations at the raphe serotonergic neurons, Tph2-ChR2(C128S) or Tph2-ChR2(C128S)::Thy1ATeam mice were unilaterally implanted with an optical fiber (⌀ 200 μm, 0.50 numerical aperture (NA), Thorlabs) into the raphe nuclei (anteroposterior (AP), −4.5 mm; mediolateral from bregma (ML), 0.0 mm; dorsoventral from the skull surface (DV), 2.5 mm) approaching with a 30-degree angle of insertion in the coronal plane (Figure S1B). We implanted the fiber for targeting the serotonergic neurons in the dorsal raphe as the primary target of photostimulation but could not exclude the possibility that the serotonergic neurons in the median raphe were also photostimulated.^72^

For fiber photometric recordings in the cortex, an optical fiber cannula (CFMC14L05, ⌀ 400 μm, 0.39 NA, Thorlabs) was unilaterally implanted into layer 5 of the right primary motor cortex (AP, +1.1 mm; ML, 1.4 mm; DV, 1.2 mm). For fiber photometric recordings with pharmacological intervention, mice were implanted with an optical fiber cannula (CFMC14L10, ⌀ 400 μm, 0.39 NA, Thorlabs) attached with a microinjection guide cannula (CXG-4T, Eicom) into the same site in the cortex.

For microinjection of AAV into the brain for fiberphotometric recordings, AAV9-hSyn-GRAB_5-HT2h_ (1.8 × 10^13^ vg per mL), AAV9-CaMKII-Laconic (5.0 × 10^13^ vg per mL), AAV9-GFAP-PinkFlamindo (1.0 × 10^13^ vg per mL), or AAV5-pZac2.1-gfABC1D-cyto-GCaMP6f (1.3 × 10^13^ vg per mL) were injected into layer 5 of the right primary motor cortex (AP, +1.1 mm; ML, 1.5 mm; DV, 1.4 mm). For microinjection of AAV into the brain for fiberphotometric control recordings, AAV10-CaMKII-CFP (1.1 × 10^13^ vg per mL), AAV10-CaMKII-mVenus (1.6 × 10^13^ vg per mL), AAV10-CaMKII-AT1.03RK (7.3 × 10^12^ vg per mL), AAV9-hSyn-EGFP (1.9 × 10^13^ vg per mL), AAV9-GFAP-GFP (1.0 × 10^14^ vg per mL), or AAV5-GFAP-mCherry (2.3 × 10^13^ vg per mL) were injected into layer 5 of the right primary motor cortex. For microinjection of AAV into the brain for inhibition of raphe serotonergic neurons, AAV8-hSyn-DIO-hM4D(Gi)-mCherry (2.0 × 10^13^ vg per mL) were injected into the dorsal raphe nuclei (AP, −4.5 mm; ML, 0.0 mm; DV, 2.5 mm) approaching with a 30-degree angle of insertion in the coronal plane. AAV microinjection was performed using a stainless-steel microinjection cannula (CXMI-4T, Eicom) attached with a 10-μL Hamilton syringe directed by a syringe pump (Legato130, KD Scientific) at a flow rate of 0.04 μL min^−1^ for 10 min. AAV microinjection was performed 3 weeks before surgery for fiber implantation.

For extracellular lactate recordings in the cortex, a guide cannula (Part 7032, Pinnacle Technology) was unilaterally implanted into the right primary motor cortex (AP, +1.1 mm; ML, 1.4 mm; DV, 1.2 mm).

All mice were implanted with electrodes for EEGs and EMGs on the skull over the frontal cortex and neck muscles, respectively, with their reference electrodes implanted on the skull over the cerebellum. To fix the heads of the mice, a U-shaped plastic plate was attached to the skull using dental cement (Fuji Lute, GC Corporation) to enable fixation to the stereotaxic frame during recordings. The mice were housed separately for a recovery period of at least 7 days.

#### Optogenetic manipulation

For photostimulation to raphe serotonergic neurons in Tph2-ChR2(C128S)::Thy1ATeam mice or Tph2-ChR2(C128S) mice, a flexible fiber-optic cable (⌀1000 μm, 0.50 NA; OPTO-LINE, Japan) was connected to an optical fiber implanted into the raphe in mice. Blue (center wavelength of 475 nm) and yellow (575 nm) light were generated by a light source (Light Engine Spectra-X; OPTO-LINE) attached to the cable. The blue and yellow light power intensities at the tip of the optical fiber were 2–3 mW and 3–4 mW, respectively. The light was controlled using a TTL pulse generator (OTPG_8, Doric lenses). TTL pulses were sent simultaneously to the light-emitting diode (LED) and the data acquisition system through an analog input to record the stimulation time with regard to fluorescent and electrical recordings from the animals. To open and close the cation-selective membrane channels of ChR2(C128S), blue and yellow light (1-s duration) were used, respectively.^72^^,^^76^ In control trials, yellow light was used instead of blue light. Photostimulation of raphe serotonergic neurons was conducted during the wake or sleep state in mice by an online visual judgment of the states with EEG and EMG signals, and the states were rejudged offline after the experiments. Serotonergic photostimulation was conducted 3 to 7 times per session, repeated at intervals of at least 5 min. For extracellular lactate recording, the intervals of photostimulation were set to more than 7 min.

#### Fiber photometry

A custom-made fiber photometric system designed by Olympus Engineering was used to detect the fluorescence of genetically encoded biosensors.^77^ For recordings of ATeam, Laconic, and control AT1.03RK and CFP signals, input light (center wavelength in 435 nm; silver-LED-435, Prizmatix) was reflected off a dichroic mirror (DM455CFP, Olympus), coupled into an optical fiber (M41L01, ⌀ 600 μm, 0.48 NA, Thorlabs), linked next to an optical fiber (M79L01, ⌀ 400 μm, 0.39 NA, Thorlabs) through a pinhole (⌀ 600 μm). It was then delivered to an optical fiber cannula (CFMC14L05, Thorlabs) implanted into the mouse brain. The LED power was <200 μW at the fiber tip. Emitted yellow and cyan fluorescence light from each probe was collected via an optical fiber cannula, divided by a dichroic mirror (DM515YFP, Olympus) into cyan (483/32 nm band-pass filter, Semrock) and yellow fluorescence (542/27 nm band-pass filter, Semrock), and detected using two distinct photomultiplier tubes (H7422-40, Hamamatsu Photonics). For the recording of control mVenus signal, input light (center wavelength of 505 nm; M505F3, Thorlabs) was used instead. For recordings of GRAB_5-HT_, GCaMP, and control GFP signals, input light (center wavelength of 475 nm; silver-LED-475, Prizmatix) and dichroic mirrors (DM490GFP, Olympus and FF552-Di02–25×36, Semrock) were used to detect green fluorescence with a band-pass filter (25BA495-540GFP, Olympus). For recordings of PinkFlamindo and control mCherry signals, input light (center wavelength of 554 nm; MINTF4, Thorlabs) and dichroic mirrors (ZT488/543rpc and T595lpxr, Chroma) were used to detect red fluorescence with a band-pass filter (ET650/100m, Chroma). Fluorescent signals were digitized using a data acquisition module (NI USB-6008, National Instruments) and recorded using a custom-made LabVIEW program (National Instruments). The signals were collected at a sampling frequency of 1 kHz. The recordings were performed under habituated head-fixed conditions during the latter half of the light phase.

#### Extracellular lactate measurements

Extracellular lactate levels were measured using a commercially available recording system (Pinnacle Technology). After a 2-week recovery period following surgery for guide implantation, the pre-calibrated lactate biosensor was inserted into the guide cannula. Recording was started after 1-h of signal stabilization. Data were collected at 1 Hz, transmitted from a potentiostat to a computer, and recorded using Sirenia Acquisition Software (Pinnacle Technology).

#### EEG and EMG recordings

EEGs and EMGs were monitored during the measurements. During fiber photometric recordings, EEG and EMG signals were amplified (Model 3000, A-M Systems), filtered, and digitized at 1 kHz using an analog-to-digital converter (USB-6008, National Instruments). EEG signals were high-pass and low-pass-filtered at 0.1 Hz and 300 Hz, respectively. The EMG signals were high-pass and low-pass filtered at 1 Hz and 300 Hz, respectively. The data acquisition software was written using LabVIEW (National Instruments). During extracellular lactate measurement, EEG and EMG signals were sampled at 1 kHz using a commercially available recording system (Pinnacle Technology).

#### Drug delivery

For microinjection of drugs to the measuring point in the cortex, a stainless-steel microinjection cannula (CXMI-4T, Eicom) with the same length as the guide cannula was inserted into the guide cannula. The microinjection cannula was connected to a 25-μL Hamilton syringe operated by an infusion pump (EP-50, Eicom) via an FEP tube (JF-10, Eicom). The following drugs were used for microinjections: 2 μM tetrodotoxin (TTX; Wako), 100 μM α-Cyano-4-hydroxycinnamic acid (4-CIN; Sigma-Aldrich), 1 mM 1,4-dideoxy-1,4-imino-D-arabinitol hydrochloride (DAB; Cayman), 500 mM sodium L-lactate (L-lac; Sigma-Aldrich), 12 mM WAY100635 (Tokyo Kasei Kogyo Co), 5 mM MDL100907 (Sigma-Aldrich), 500nM SB204741 (Sigma-Aldrich), 5 mM SB242084 (Cayman), 26 mM scopolamine (Sco; Tokyo Kasei Kogyo Co), 1 mM mecamylamine hydrochloride (MEC; Cayman) and 0.11 mM methyllycaconitine citrate (MLA; Cayman). Drugs were dissolved in the vehicle (HEPES-Ringer’s solution): 125 mM NaCl, 5 mM KCl, 10 mM HEPES, 2 mM CaCl_2_, 2 mM MgSO_4_, and 10 mM glucose, pH 7.4. For 4-CIN and SB204741, drugs were dissolved in 1% and 0.001% dimethylsulfoxide (DMSO)-containing HEPES-Ringer’s solution, respectively. MDL100907 was dissolved in a 10% 2-hydroxypropyl-b-cyclodextrin (HBC)-containing HEPES-Ringer’s solution. The microinjection volume was 0.2 μL, administered at a rate of 0.04 μL/min. After a 15-min post-injection period, the measurement resumed.

For intraperitoneal administration, the mice were injected with fluoxetine (5 mg/kg body weight; Tocris Bioscience) or scopolamine (1 mg/kg body weight; Tokyo Kasei Kogyo). After a 15-min post-injection period, the measurement resumed. To lesion the central noradrenergic system, mice were intraperitoneally injected with N-(2-chloroethyl)-N-ethyl-2-bromobenzylamine (DSP-4; Tocris). Seven days after the first injection (60 mg/kg body weight), a second injection was administered (50 mg/kg body weight).^78^^,^^79^ Ten days after the first injection, the measurement was resumed. For chemogenetic inhibition of raphe serotonergic neurons, clozapine-N-oxide (CNO; Tocris Bioscience) dissolved in saline solution to a concentration of 0.3 mg/mL was used. The CNO (3 mg/kg body weight) or saline was administered to each mouse intraperitoneally 1 hour before initiating the Thy1-ATeam recording, which was repeatedly performed at intervals of at least 1 week.

#### Immunohistochemistry

Mice were deeply anesthetized with a ketamine-xylazine mixture (100 and 10 mg/kg body weight, respectively, intraperitoneally) and perfused with 4% paraformaldehyde in 0.1 M phosphate-buffer (PB). The brains were removed from the skull and post-fixed in the same fixative overnight. Subsequently, the brains were cryoprotected in 20% sucrose/PB overnight, frozen, and cut to a thickness of 50 μm on a cryostat. The sections were rinsed with phosphate-buffered saline containing 0.1% Triton X-100 (PBST) and incubated with primary antibodies overnight at room temperature. The following antibodies were used: goat anti-GFP (1:250, #600-101-215, Rockland), mouse anti-CaMKII (1:200, ab22609, Abcam), rabbit anti-GFAP (1:1000, G9269, Sigma-Aldrich), mouse anti-GFAP (1:1000, G3893, Sigma-Aldrich), and rabbit anti-DsRed (1:1000, #632496, Clontech). For fluorescence microscopy, the sections were treated with a mixture of species-specific secondary antibodies conjugated to AlexaFluor-488 or -594 (1:500, Thermo Fisher Scientific) for 2 hat room temperature. Fluorescence images were obtained using a fluorescence microscope (BZ-X800, Keyence).

### Quantification and statistical analysis

#### Fiber photometric and pinnacle data analysis

Under serotonergic photostimulation, FRET-based ratiometric Thy1-ATeam and CaMKII-Laconic signals (mVenus/CFP and CFP/mVenus fluorescence intensities, respectively), filtered with a 100-point moving average, were shown as ΔR/R by calculating (R – R0) / R0, where R0 is the averaged value of the signals for 5 s just before the light illumination. Other fiber-photometric fluorescent signals were filtered with a 100-point moving average and shown as ΔF/F by calculating (F – F0) / F0, where F0 is the averaged value of the signals for 5 s just before the light illumination. The signal under serotonergic photostimulation was averaged in each session, consisting of more than 3 repetitive stimulations for each mouse, and was dealt with as individual data and used for making averaged signal traces and for quantitative analysis. For calculating the quantitative parameters such as peak values and AUCs, the individual fluorescent signal datafurther smoothed by a 500-point moving average was used. The AUC of fluorescent signal responses to serotonergic activation was calculated from the data for 30 s of light stimulation, except for 1 s each of the first (blue) and second (yellow) light exposures, unless otherwise stated. Cortical extracellular lactate levels were normalized by the mean values for 5 s just before the light illumination as 100%. The extracellular lactate signal was quantitatively evaluated with the data in two phases: 0–30 s (during the light illumination) and 30–400 s (after the light illumination), respectively. For the analysis of the Thy1-ATeam signal within the vigilance state transitions, ΔR/R was calculated by (R – R0) / R0, where R0 is the averaged value of the signal for 5 s immediately before the transitions. In the case of sub-state transitions, the Thy1-ATeam signal averaged across all identified onsets exhibited by each animal was adjusted to a zero point with the mean values of one epoch (4 s) immediately before the onset time. The signals aligned to the state/sub-state transition were averaged in each session, defined as the 30-minute measurement with at least one event occurring for each mouse, dealt with as individual data, and used for making averaged signal traces and for quantitative analysis. These fiber photometric and pinnacle data analyses were performed using custom MATLAB (MathWorks) code (Data S1, related to Figures 1,2,3,4,5, and 6).

#### Vigilance state determination

EEG/EMG recordings were automatically scored offline as wakefulness, non-REM sleep, or REM sleep state using SleepSign software version 3 (Kissei Comtec) in 4-s epochs according to standard criteria.^80^ Then, all vigilance state classifications assigned by SleepSign were virtually modified to be scored every second to compare the signal at the time of serotonergic photostimulation-induced awakening and spontaneous awakening of mice in seconds. The same individual, blinded to the genotype and experimental conditions, scored all EEG/EMG recordings. To detect the onset of active-awake in the wake state and micro-awakening in the NREM sleep state, the time point at which EMG activity was above a threshold value (mean greater than 10 times the standard deviation) was obtained within each major state.^12^

#### Statistics

The statistics were calculated using MATLAB software (MathWorks). The stimulation effects of *in vivo* optogenetics and fluorescent signals to examine them are influenced by the condition of the optical fiber implanted in animals, which varies among individual animals. Since these data were not normally distributed, we performed non-parametric tests for statistical analysis. Two-sample comparisons were performed using the two-sided Wilcoxon signed-rank test or two-sided Mann–Whitney *U*-test. Multiple group comparisons of break points were performed using the Friedman test and Kruskal-Wallis test, followed by the post hoc Steel-Dwass test or Steel test. Data with error bars represent the mean ± SEM in each graph unless otherwise stated. All the tests are specified in the figure legends.

## Data Availability

Data reported in this paper will be shared by the lead contact upon request. All original code is available in this paper’s supplemental information. Any additional information required to reanalyze the data reported in this paper is available from the lead contact upon request.

## Supplemental information

Document S1. Figures S1–S7

Data S1. Matlab Codes, related to STAR Methods
