# Supplementary material for: Serotonergic neurons control cortical neuronal intracellular energy dynamics by modulating astrocyte-neuron lactate shuttle
Source: iScience. 2023 Jan 5;26(1):105830. doi: 10.1016/j.isci.2022.105830 (PMC9881222; doi:10.1016/j.isci.2022.105830)
Supplement: Document S1. Figures S1–S7 [file mmc1.pdf]

iScience, Volume *26*

Supplemental information

Serotonergic neurons control cortical neuronal intracellular energy dynamics by modulating astrocyte-neuron lactate shuttle

Akiyo Natsubori, Shinobu Hirai, Soojin Kwon, Daisuke Ono, Fei Deng, Jinxia Wan, Momoka Miyazawa, Takashi Kojima, Haruo Okado, Akihiro Karashima, Yulong Li, Kenji F. Tanaka, and Makoto Honda

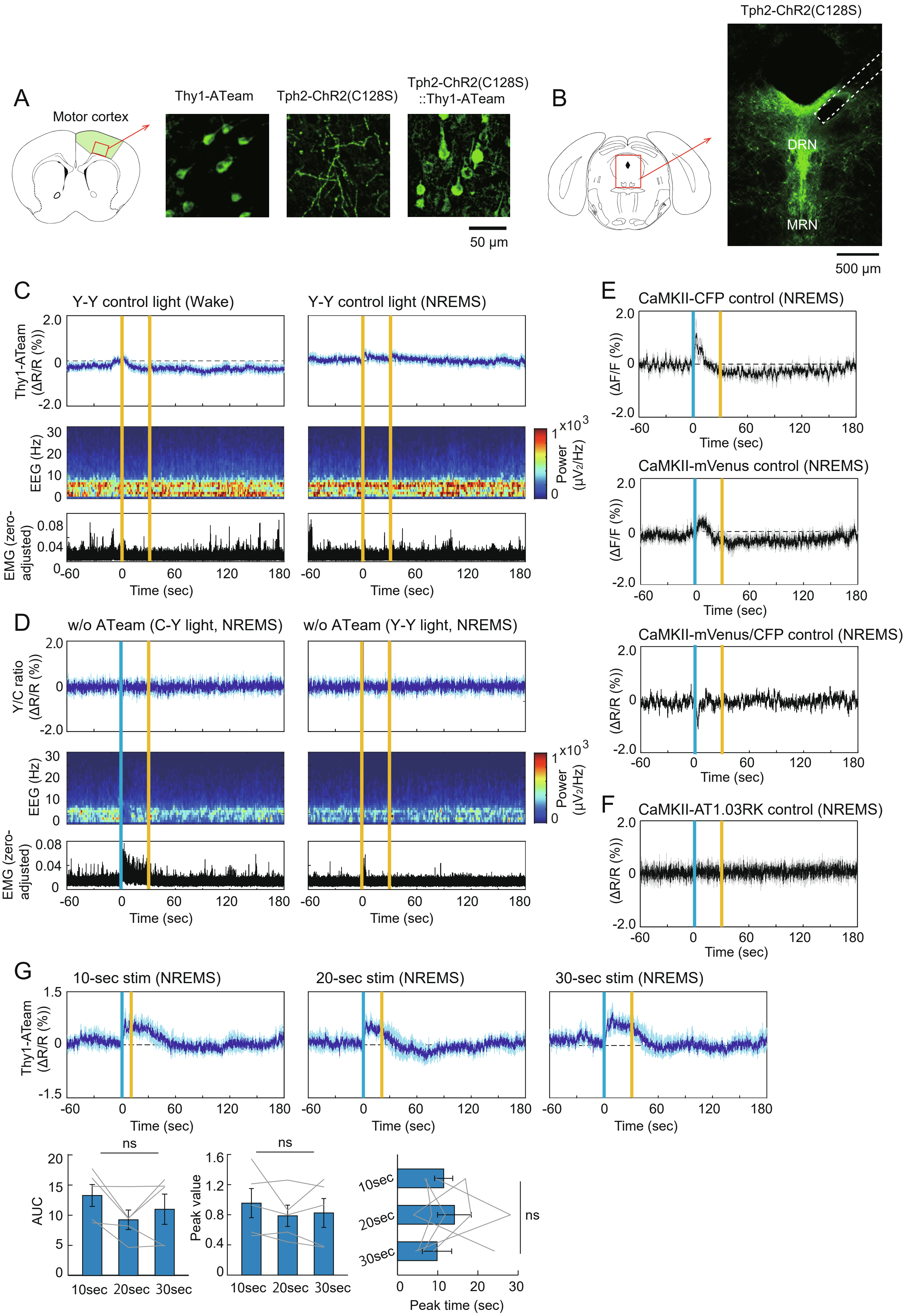

A
Motor cortex
Thy1-ATeam
Tph2-ChR2(C128S)
Tph2-ChR2(C128S)
::Thy1-ATeam
50 μm
B
Tph2-ChR2(C128S)
DRN
MRN
500 μm
C
Y-Y control light (Wake)
Y-Y control light (NREMS)
Thy1-ATeam (ΔR/R (%))
EEG (Hz)
EMG (zero-adjusted)
Power (μV2/Hz)
x10^3
Time (sec)
D
w/o ATeam (C-Y light, NREMS)
w/o ATeam (Y-Y light, NREMS)
Y/C ratio (ΔR/R (%))
E
CaMKII-CFP control (NREMS)
CaMKII-mVenus control (NREMS)
CaMKII-mVenus/CFP control (NREMS)
(ΔF/F (%))
(ΔR/R (%))
F
CaMKII-AT1.03RK control (NREMS)
G
10-sec stim (NREMS)
20-sec stim (NREMS)
30-sec stim (NREMS)
AUC
Peak value
Peak time (sec)
ns
10sec
20sec
30sec

Fig. S1 Characterization of cortical neuronal intracellular ATP signal measurements under raphe serotonergic photostimulations (related to Fig. 1).

(A) GFP fluorescence images in layer 5 of the motor cortex of Thy1-ATeam (left), Tph2-ChR2(C128S) (middle), and Tph2-ChR2(C128S)::Thy1-ATeam mice (right). Green represents GFP staining. Scale bar, 50 µm. In Thy1-ATeam mice, ATeam expression in pyramidal neurons is exhibited by GFP immunostaining. In Tph2-ChR2(C128S) mice, ChR2 expression in axon terminals of presumably serotonergic neurons is exhibited by GFP immunostaining. In Tph2-ChR2(C128S)::Thy1-ATeam mice, both ATeam-positive pyramidal neurons and ChR2-positive serotonergic axon terminals were closely distributed in the cortex exhibited as GFP-positive. (B) Histological reconstruction of optical fiber tip placement in the DRN of Tph2-ChR2(C128S) mice. Green represents GFP staining. Scale bar, 500 µm. DRN, dorsal raphe nucleus; MRN, median raphe nucleus. The atlas templates are from the atlas of Paxinos and Franklin (2004). (C) Traces of averaged Thy1-ATeam signal fluctuations, EEG power density spectrum, and EMG activity under the control yellow light stimulation to raphe serotonergic neurons during the wake (left) and NREM sleep states (right) in Tph2-ChR2::Thy1-ATeam mice. Vertical yellow lines indicate the 1-s light illuminations. Traces of Thy1-ATeam signals represent mean ± SEM (n = 5 sessions from 5 mice; same as Fig. 1). Refer to Fig. 1D for the corresponding analysis. (D) Traces of averaged yellow/cyan fluorescence intensity ratio (Y/C ratio) fluctuations in Tph2-ChR2(C128S) mice (w/o: without ATeam) under serotonergic photostimulations (left) and control yellow light illuminations (right) during the NREM sleep states, respectively (n = 3 session from 3 mice). Vertical blue and yellow lines indicate 1-s illumination of each light color. Note that the optogenetic activation of raphe serotonergic neurons induced the change of EEG/EMG signals, but not that of the cortical fluorescent signals without ATeam probes. (E) Traces of averaged CaMKII-CFP (top) and CaMKII-mVenus (middle) fluorescence intensity fluctuations and their ratio (mVenus/CFP; bottom) under serotonergic photostimulations. Traces of CaMKII-CFP and CaMKII-mVenus signals represent mean ± SEM (n = 5 sessions from 5 mice). (F) Traces of averaged CaMKII-AT1.03RK (ATeam mutant probe) signal fluctuation under serotonergic photostimulations. Traces of signals represent mean ± SEM (n = 3 sessions from 3 mice). (G) Top: Traces of Thy1-ATeam signal responses to serotonergic photostimulations for 10, 20, and 30 s during the NREM sleep states, respectively. Traces of signals represent mean ± SEM (n = 5 sessions from 5 mice; same as Fig. 1). Bottom: Comparison of Thy1-ATeam signal responses to serotonergic photostimulation of different duration. Friedman test (n = 5 sessions from 5 mice). Data are expressed as mean ± SEM.

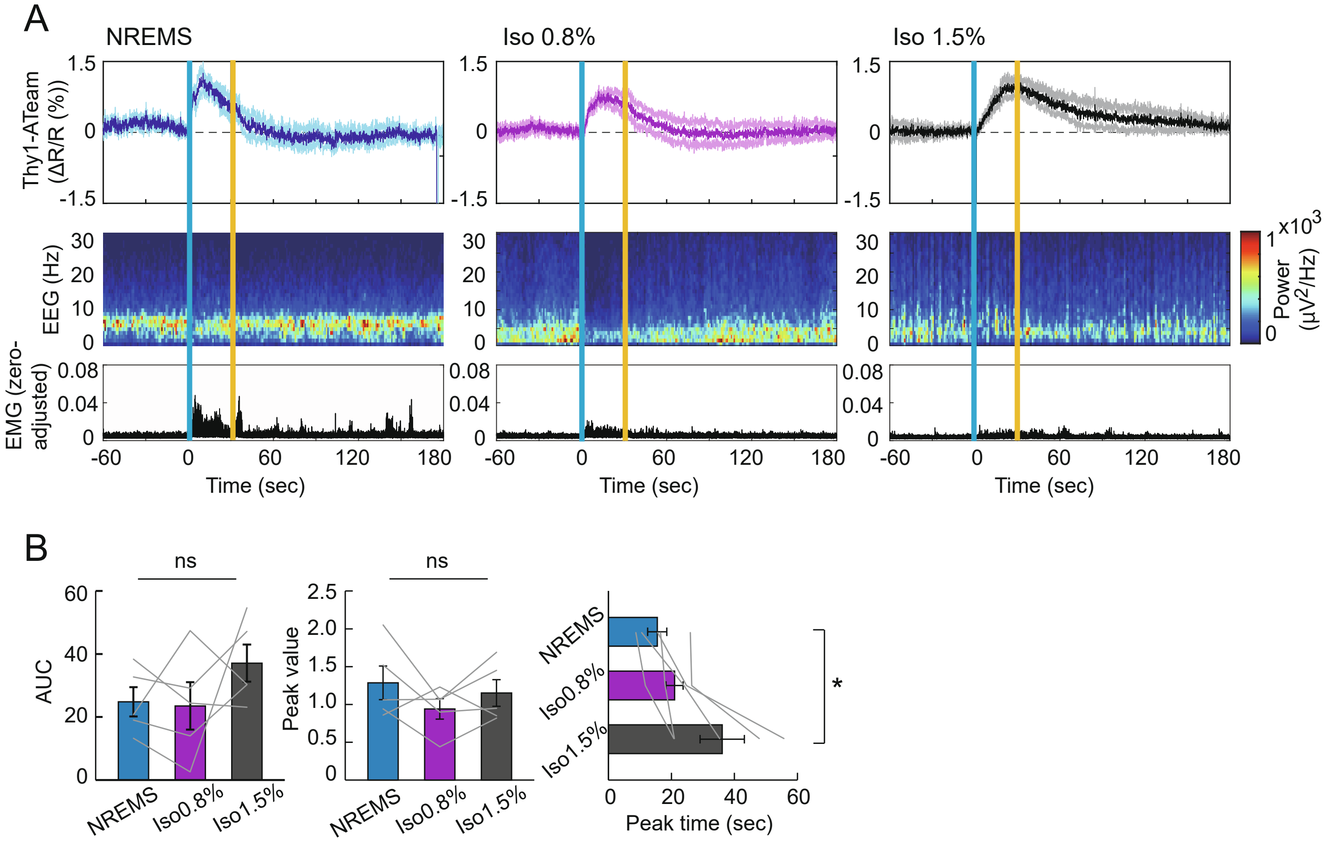
A
NREMS
Iso 0.8%
Iso 1.5%
Thy1-ATeam (ΔR/R (%))
1.5
0
-1.5
EEG (Hz)
30
20
10
0
x10^3
1
0
Power (μV²/Hz)
EMG (zero-adjusted)
0.08
0.04
0
-60
0
60
120
180
Time (sec)
B
ns
AUC
60
40
20
0
NREMS
Iso0.8%
Iso1.5%
Peak value
2.5
2.0
1.5
1.0
0.5
0
Peak time (sec)
*

Fig. S2 Slower response of cortical neuronal intracellular ATP signals to raphe serotonergic activations under isoflurane anesthesia (related to Fig. 1).

(A) Traces of averaged Thy1-ATeam signal fluctuations, EEG power density spectrum, and EMG activities under serotonergic photostimulations for 30 s during the NREM sleep states (left) and under 0.8% (middle) and 1.5% isoflurane anesthesia (right), respectively. Vertical blue and yellow lines indicate 1-s illumination of each light color. Traces of Thy1-ATeam signals represent mean ± SEM (n = 5 sessions from 5 mice; same as Fig.1). (B) Comparison of Thy1-ATeam signal responses to serotonergic photostimulation during the NREM sleep and under the distinct concentrations of isoflurane anesthesia. *$p < 0.05$ vs. NREMS; Friedman test with *post hoc* Steel test (n = 5 sessions from 5 mice). Data are expressed as mean ± SEM.

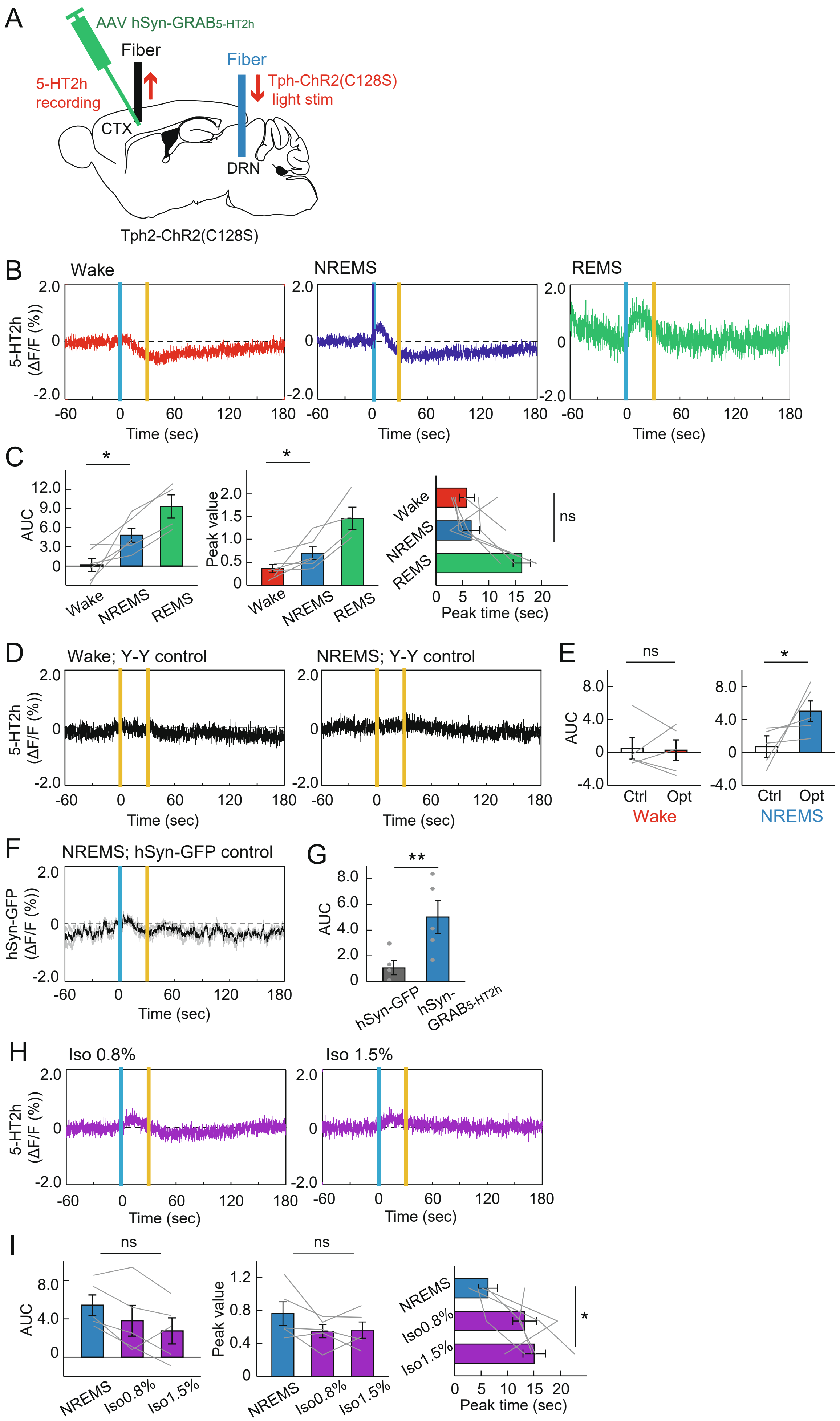
A
AAV hSyn-GRAB5-HT2h
Fiber
5-HT2h recording
Fiber
Tph-ChR2(C128S) light stim
CTX
DRN
Tph2-ChR2(C128S)
B
Wake
NREMS
REMS
5-HT2h (ΔF/F (%))
Time (sec)
C
AUC
Peak value
Peak time (sec)
Wake
NREMS
REMS
ns
D
Wake; Y-Y control
NREMS; Y-Y control
E
ns
Ctrl
Opt
Wake
NREMS
F
NREMS; hSyn-GFP control
hSyn-GFP (ΔF/F (%))
G
**
hSyn-GFP
hSyn-GRAB5-HT2h
H
Iso 0.8%
Iso 1.5%
I
Iso0.8%
Iso1.5%

Fig. S3 State-dependent cortical extracellular serotonin signal responses to raphe serotonergic activations (related to Fig. 1).

(A) Schematic illustration of fiber photometric recording of extracellular serotonin levels in the cortex under the raphe serotonergic photostimulation in Tph2-ChR2(C128S) mice injected with AAV-hSyn-GRAB$_{5\text{-}HT2h}$. CTX, cortex; DRN, dorsal raphe nucleus. (B) Traces of averaged hSyn-GRAB$_{5\text{-}HT2h}$ signals under serotonergic photostimulation during the wake, NREM sleep, and REM sleep states, respectively (n = 6 sessions from 4 mice for wake and NREM sleep; n = 4 sessions from 3 mice for REM sleep). Vertical blue and yellow lines indicate 1-s illumination of each light color. (C) Comparison of AUC, peak value, and the peak time of hSyn-GRAB$_{5\text{-}HT2h}$ signal responses to the serotonergic activation across the states for the data in (B). *$p < 0.05$: Wake vs. NREMS; two-sided Wilcoxon signed-rank test (n = 6 sessions from 4 mice). The data in REM sleep were excluded from the statistical analysis due to a lack of sample size. (D) Traces of averaged hSyn-GRAB$_{5\text{-}HT2h}$ signals under the control yellow light illumination to raphe serotonergic neurons during the wake (left) and NREM sleep states (right), respectively (n = 5 sessions from 4 mice). (E) AUC of hSyn-GRAB$_{5\text{-}HT2h}$ signal responses to the optogenetic activation of serotonergic neurons. *$p < 0.05$ vs. Control; two-sided Wilcoxon signed-rank test (n = 5 sessions from 4 mice). (F) Traces of averaged control hSyn-GFP fluorescence intensity fluctuation under serotonergic photostimulation (n = 5 sessions from 4 mice). (G) Comparison of hSyn-GFP and hSyn-GRAB$_{5\text{-}HT2h}$ fluorescent fluctuations under serotonergic photostimulation. **$p < 0.01$ vs. hSyn-GFP; Mann–Whitney test (n = 5 sessions from 4 mice, respectively). (H) Traces of averaged hSyn-GRAB$_{5\text{-}HT2h}$ signal responses to serotonergic photostimulations under the 0.8% (left) and 1.5% isoflurane anesthesia (right), respectively (n = 5 sessions from 3 mice). (I) Comparison of hSyn-GRAB$_{5\text{-}HT2h}$ signal responses to serotonergic photostimulation during the NREM sleep and under the distinct concentrations of isoflurane anesthesia. *$p < 0.05$ vs. NREMS; Kruskal–Wallis test (n = 5 sessions from 3 mice). Data are expressed as mean ± SEM.

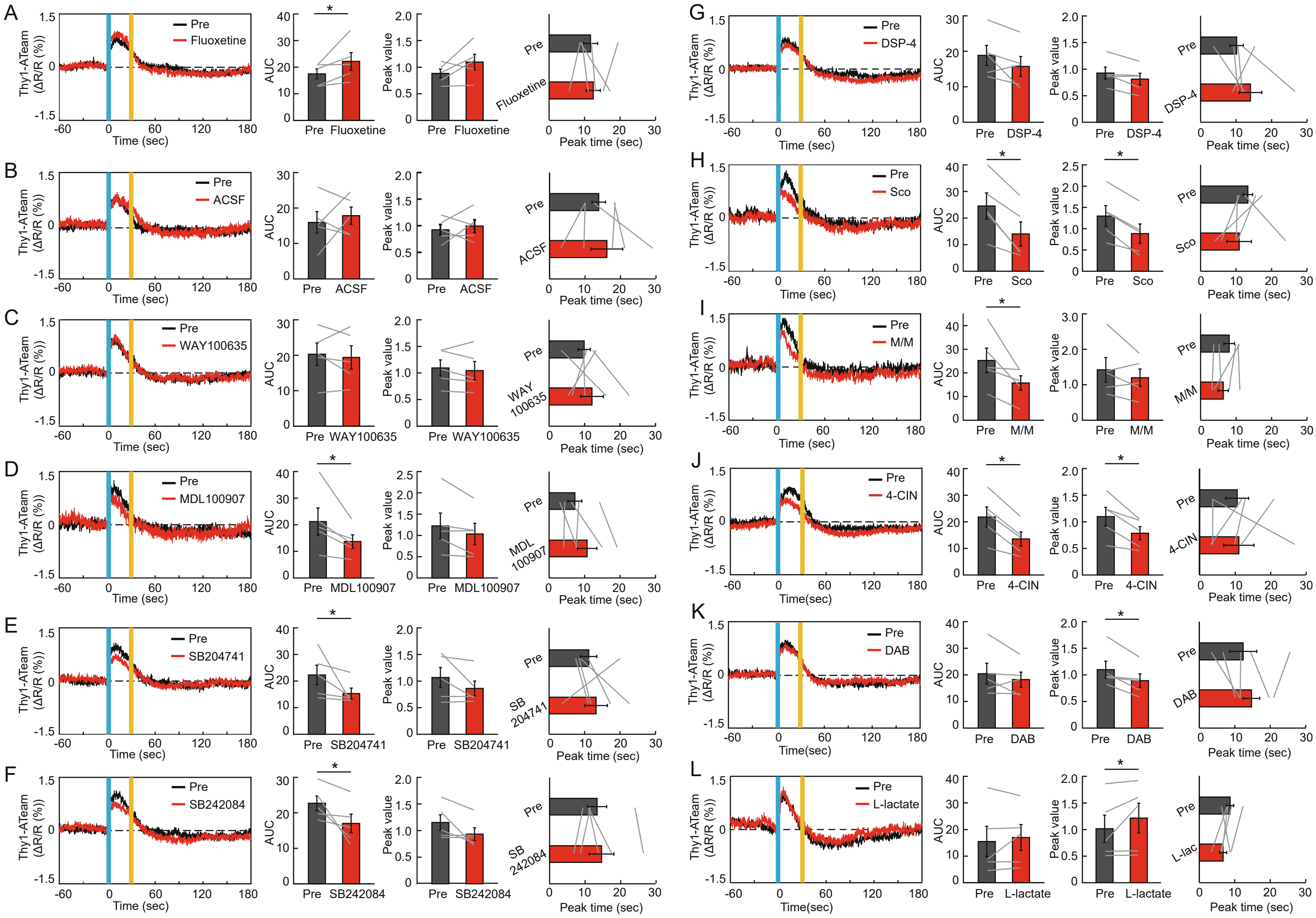
A
B
C
D
E
F
G
H
I
J
K
L
Thy1-ATeam (ΔR/R (%))
Time (sec)
AUC
Peak value
Peak time (sec)
Pre
Fluoxetine
ACSF
WAY100635
MDL100907
SB204741
SB242084
DSP-4
Sco
M/M
4-CIN
DAB
L-lactate
L-lac

Fig. S4 Pharmacological effects on cortical neuronal intracellular ATP signal response to serotonergic photostimulation (related to Figs. 1 and 2).

Comparison of Thy1-ATeam signal responses to serotonergic photostimulation before and after treatment with fluoxetine (A), ACSF (B), WAY100635 (C), MDL100907 (D), SB204741 (E), SB242084 (F), DSP-4 (G), scopolamine (Sco) (H), mecamylamine/methyllycaconitine (M/M) (I), 4-CIN (J), DAB (K), and L-lactate (L). The averaged signal traces (far left) and alterations of the AUC (left), peak value (right), and peak time (far right) under serotonergic photostimulation by each drug treatment are shown as additional data in Figs. 1H1-J and 2. The averaged signal traces in (J), (K), and (L) are consistent with Fig. 2B, 2E, and 2H, respectively. *$p < 0.05$ vs. Pre (before the treatment); two-sided Wilcoxon signed-rank test (n = 5 sessions from 5 mice). Vertical blue and yellow lines indicate the 1-s illumination of each light color. Data are expressed as the mean ± SEM.

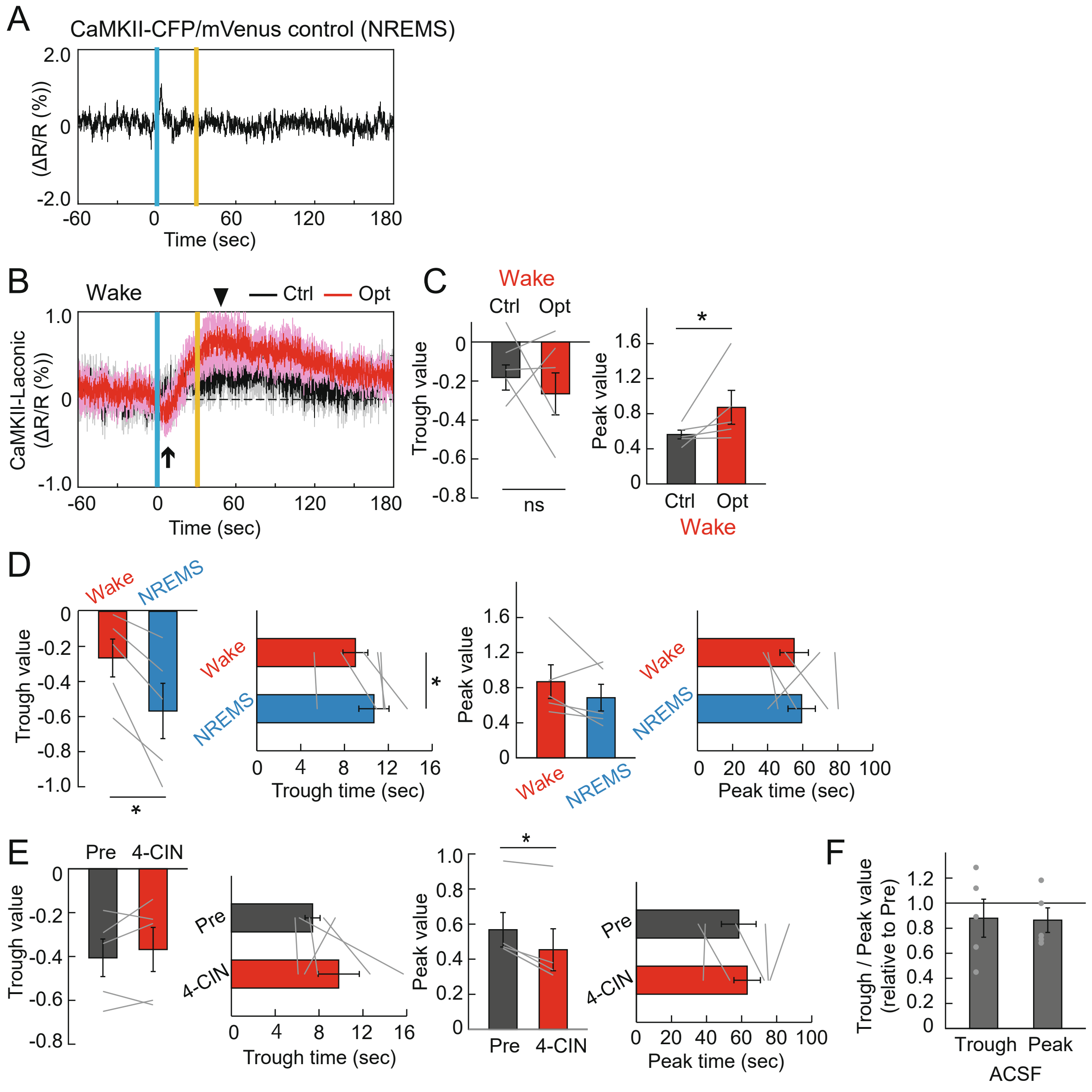
A
CaMKII-CFP/mVenus control (NREMS)
(ΔR/R (%))
Time (sec)
B
Wake
Ctrl
Opt
CaMKII-Laconic (ΔR/R (%))
Time (sec)
C
Wake
Ctrl
Opt
Trough value
ns
Peak value
*
Ctrl
Opt
Wake
D
Wake
NREMS
Trough value
*
Trough time (sec)
Peak value
Peak time (sec)
E
Pre
4-CIN
Trough value
Trough time (sec)
Peak value
*
Peak time (sec)
F
Trough / Peak value (relative to Pre)
Trough
Peak
ACSF

Fig. S5 Effect of state and drug-microinjection on cortical neuronal intracellular lactate signal responses to serotonergic photostimulation (related to Fig. 3).

(A) Traces of averaged ratio of CaMKII-CFP to CaMKII-mVenus fluorescence intensity fluctuations under serotonergic photostimulation (n = 5 sessions from 5 mice). The presented data were calculated from the data in Fig. S1E. Vertical blue and yellow lines indicate 1-s illumination of each light color. (B) Traces of CaMKII-Laconic signals in the cortex under serotonergic photostimulation (Opt) and control light illuminations (Ctrl) during the wake state. In the control condition, yellow light illumination was used in place of blue light. Traces of CaMKII-Laconic signals represent mean ± SEM (n = 5 sessions from 5 mice; same as Fig. 3). (C) Initial trough (arrow in (B)) and subsequent peak (arrowhead in (B)) value of CaMKII-Laconic signal responses to serotonergic photostimulation during the wake state of mice. *$p < 0.05$ vs. Control; two-sided Wilcoxon signed-rank test (n = 5 sessions from 5 mice). Note that the temporal decrease of the CaMKII-Laconic signal was not evident by serotonergic photostimulation during the wake state. (D) Comparison of CaMKII-Laconic signal responses to serotonergic photostimulation during the wake and NREM sleep states, for the data in Fig. 3C. *$p < 0.05$ vs. Wake; two-sided Wilcoxon signed-rank test (n = 5 sessions from 5 mice). (E) Effect of 4-CIN administration on the peak/trough values and their timings of CaMKII-Laconic signal under serotonergic photostimulation was shown as additional data in Fig. 3H. *$p < 0.05$ vs. Pre; two-sided Wilcoxon signed-rank test (n = 5 sessions from 5 mice). (F) No effect of ACSF microinjection on CaMKII-Laconic signal responses to serotonergic photostimulation. Two-sided Wilcoxon signed-rank test (n = 5 sessions from 5 mice). Data are expressed as mean ± SEM.

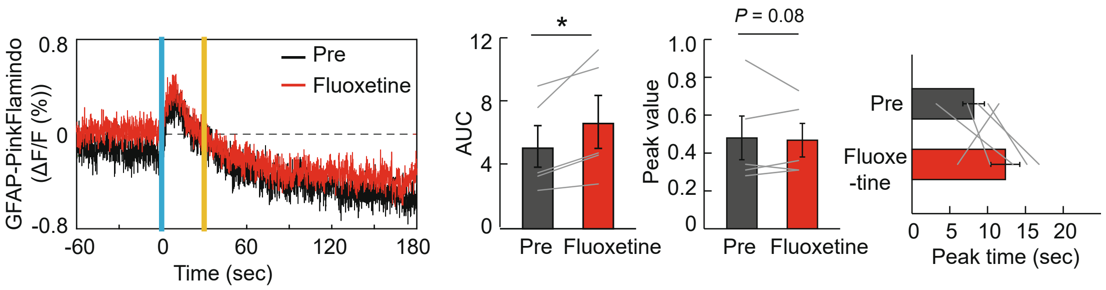

GFAP-PinkFlamindo (ΔF/F (%))
0.8
0
-0.8
-60
0
60
120
180
Time (sec)
Pre
Fluoxetine
*
12
8
4
0
AUC
Pre
Fluoxetine
P = 0.08
1.0
0.8
0.6
0.4
0.2
0
Peak value
Pre
Fluoxetine
Pre
Fluoxe
-tine
0
5
10
15
20
Peak time (sec)

Fig. S6 Cortical astrocytic cAMP signal response to serotonergic photostimulation was affected by the treatment with fluoxetine (related to Fig. 5).

Comparison of the GFAP-PinkFlamindo signal responses to serotonergic photostimulation before and after treatment with fluoxetine. The averaged signal traces (far left) and alteration of the AUC (left), peak value (right), and peak time (far right) under serotonergic photostimulation by the fluoxetine treatment are shown as additional data in Fig. 5F. $*p < 0.05$ vs. Pre (before the treatment); two-sided Wilcoxon signed-rank test (n = 5 sessions from 5 mice). Vertical blue and yellow lines indicate the 1-s illumination of each light color. Data are expressed as the mean ± SEM.

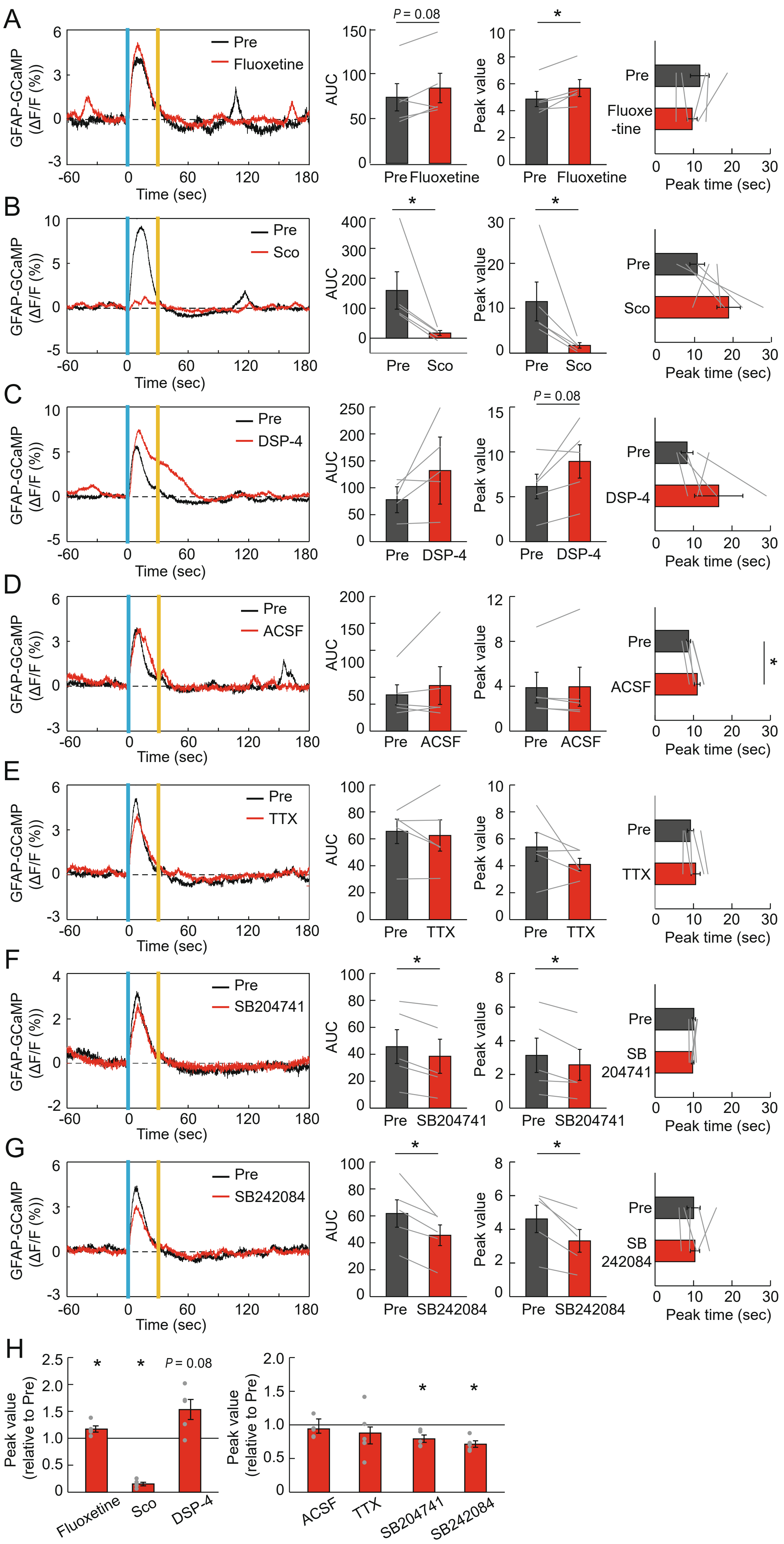
A
B
C
D
E
F
G
H
GFAP-GCaMP (ΔF/F (%))
Time (sec)
Pre
Fluoxetine
Sco
DSP-4
ACSF
TTX
SB204741
SB242084
AUC
Peak value
Peak time (sec)
P = 0.08
*
Fluoxe -tine
SB 204741
SB 242084
Peak value (relative to Pre)

Fig. S7 Cortical astrocytic Ca^{2+} signal response to serotonergic photostimulation was affected by treatment with scopolamine and DSP-4, as well as the serotonin-related drugs (related to Fig. 5)

Comparison of the GFAP-GCaMP signal responses to serotonergic photostimulation before and after treatment with fluoxetine (A), scopolamine (Sco) (B), DSP-4 (C), ACSF (D), TTX (E), SB204741 (F), and SB242084 (G). The averaged signal traces (far left) and alteration of the AUC (left), peak value (right), and peak time (far right) under serotonergic photostimulation by each drug treatment are shown as additional data in Fig. 5J-K. Vertical blue and yellow lines indicate the 1-s illumination of each light color. (H) The effect of fluoxetine, scopolamine, and DSP-4 treatment (left) and ACSF, TTX, SB204741, and SB242084 microinjection (right) on the peak values of the GFAP-GCaMP signal responses to serotonergic photostimulation. *$p < 0.05$ vs. Pre (before the treatment); two-sided Wilcoxon signed-rank test (n = 5 sessions from 5 mice). Data are expressed as the mean ± SEM.
