# Supplementary material for: Serotonergic neurons control cortical neuronal intracellular energy dynamics by modulating astrocyte-neuron lactate shuttle
Source: iScience. 2023 Jan 5;26(1):105830. doi: 10.1016/j.isci.2022.105830 (PMC9881222; doi:10.1016/j.isci.2022.105830)
Supplement: Data S1. Matlab Codes, related to STAR Methods [file mmc2.zip › Procedure_for_using_MatlabCodes.docx]

The Matlab codes presented in Data S1 are used to display and analyze the fiber photometric and pinnacle data.

The procedure for using the codes in the fluorescent signal analysis is as follows:

1. To display filtered fluorescent signals (ATeam, Laconic, GRAB_5-HT_, GCamp, PinkFlamindo, and their control probe signals) and simultaneously recorded EEG, EMG, and TTL signals, run the subplot_allsignals.m-file in MATLAB. Note that readmulti_DBL.m-file is required to run this m-file.
2. Run the TTL_trig_ses.m-file to align the fluorescent signal data in a session at a specific time (e.g., the onset time of serotonergic photostimulation) identified by the TTL signal and obtain the averaged signal.
3. Execute the AUC_response.m-file to calculate the AUC of the fluorescent signal per session.
4. Execute the TTL_trig_merge.m-file to obtain the averaged signal traces across sessions.
